# Orderly mitosis shapes interphase genome architecture

**DOI:** 10.1101/2025.06.03.657645

**Published:** 2025-06-03

**Authors:** Krishnendu Guin, Adib Keikhosravi, Gianluca Pegoraro, Raj Chari, Tom Misteli

**Affiliations:** 1 Cell Biology of Genomes, National Cancer Institute, NIH, Bethesda, MD 20892, USA; 2 High Throughput Imaging Facility (HiTIF), National Cancer Institute, NIH, Bethesda, MD 20892, USA; 3 Genome Modification Core (GMC), Frederick National Lab for Cancer Research, Frederick, MD 21702, USA

**Keywords:** centromere, 3D genome organization, high throughput imaging, cell cycle, mitosis, functional genomics screening, CRISPR

## Abstract

Genomes assume a complex 3D architecture in the interphase cell nucleus. Yet, the molecular mechanisms that determine global genome architecture are only poorly understood. To identify mechanisms of higher order genome organization, we performed high-throughput imaging-based CRISPR knockout screens targeting 1064 genes encoding nuclear proteins in human cell lines. We assessed changes in the distribution of centromeres at single cell resolution as surrogate markers for global genome organization. The screens revealed multiple major regulators of spatial distribution of centromeres including components of the nucleolus, kinetochore, cohesins, condensins, and the nuclear pore complex. Alterations in centromere distribution required progression through the cell cycle and acute depletion of mitotic factors with distinct functions altered centromere distribution in the subsequent interphase. These results identify molecular determinants of spatial centromere organization, and they show that orderly progression through mitosis shapes interphase genome architecture.

## Introduction

Genomes are complex polymers. In the cell nucleus, the genome is organized via several structures at different length-scales. At the shortest scale, genomic DNA is wrapped around a histone octamer to form a nucleosome (Wen, Fang et al. 2025). Strings of nucleosomes then fold onto themselves to form a chromatin fiber, which in turn organizes itself into chromatin loops, typically in the 1–100 kb range. Chromatin fibers further fold into 0.5–2 Mb topologically associated domains (TADs), which can homotypically associate with each other into transcriptionally active A compartments and transcriptionally repressed B compartments spanning several megabases (Rowley and Corces 2018, Hildebrand and Dekker 2020, Misteli 2020, Paldi and Cavalli 2025). While these genome features occur in most cell types and species, all chromatin features exhibit extensive single-cell variability (Finn and Misteli 2019).

The organization of genomes is non-random within the cell nucleus (Parada and Misteli 2002, Oliver and Misteli 2005, Bouwman, Crosetto et al. 2022). Chromosomes and individual gene loci tend to occupy preferred positions relative to the nuclear boundary and relative to each other (Shachar and Misteli 2017, Scholz, Sumida et al. 2019, Bouwman, Crosetto et al. 2022). For example, the chromosomes that contain clusters of ribosomal genes congregate in 3D space to form the subnuclear compartment of the nucleolus (Dundr, Misteli et al. 2000). Similarly, transcriptionally repressive genome regions are often associated with the nuclear lamina at the periphery of the cell nucleus and around the nucleolus (Croft, Bridger et al. 1999, Alagna, Thomas et al. 2023). Defects in spatial genome organization are associated with multiple diseases including cancer and accelerated aging (Misteli 2010, Wang, Luo et al. 2023, Amodeo, Eyler et al. 2025).

Recent studies have shed light on to the mechanisms that determine the local organization of the genome (Bonev and Cavalli 2016, Finn and Misteli 2019, Misteli 2020). Chromatin loops and domains are formed by loop extrusion, in which the ringlike condensin protein complex acts as a molecular motor (Terakawa, Bisht et al. 2017) to extrude the chromatin fiber to form loops, which can eventually congregate into TADs (Ganji, Shaltiel et al. 2018, Davidson and Peters 2021). In contrast, the molecular mechanisms that determine the higher-order global genome organization, such as the location of genes, chromatin domains or chromosomes within the 3D space of the nucleus, are less clear. Some insights come from the observation in yeast and *C. elegans* that transcriptionally repressive chromosomes are preferentially tethered to the nuclear periphery via histone modifications (Towbin, Gonzalez-Aguilera et al. 2012, Shachar and Misteli 2017). Furthermore, unbiased screening approaches have suggested that progression through S-phase is essential to establish the nuclear location of individual genes (Joyce, Williams et al. 2012, Shachar, Voss et al. 2015). It has also been suggested that the propensity to undergo homotypic interactions promotes the clustering of similar genome regions, for example, the association of ribosomal genes in the nucleolus (Lafontaine, Riback et al. 2021) or the formation of intranuclear heterochromatin blocks (Misteli 2020).

The centromere is a prominent structural feature of all chromosomes (Murray and Szostak 1985). Centromeres are specialized genomic loci that assemble the kinetochore protein complex, which connects chromosomes with the microtubule spindle during mitosis, and through their attachment ensure error-free chromosome segregation (McKinley and Cheeseman 2016). Like other chromosomal features, centromeres have been observed to assume non-random locations in the cell nucleus across species. In yeast, centromeres cluster and localize at the nuclear periphery at some or all stages of the cell cycle (Guin, Sreekumar et al. 2020). Variable degrees of clustering have been observed in apicomplexan parasites (Bunnik, Venkat et al. 2019), plants (Fransz, De Jong et al. 2002), flies (Padeken, Mendiburo et al. 2013), and mice (Weierich, Brero et al. 2003, Stevens, Lando et al. 2017), where centromeres cluster into prominent chromocenters, presumably via homotypic interactions (Brandle, Fruhbauer et al. 2022). In humans, centromere clustering is less pronounced but increased clustering of centromeres near nucleoli has been observed in multiple cell lines (Weierich, Brero et al. 2003, Bury, Moodie et al. 2020, Rodrigues, MacQuarrie et al. 2023, Kumar, Gholamalamdari et al. 2024), particularly prominently in human stem cells where most centromeres are localized near nucleoli (Wiblin, Cui et al. 2005, Rodrigues, MacQuarrie et al. 2023). The fact that clustered centromeres tend to dissociate from the nucleolus during stem cell differentiation (Rodrigues, MacQuarrie et al. 2023) may point to a functional role of nucleolar centromere clustering. However, the underlying molecular mechanisms determining spatial centromere distribution remain elusive.

Given their prominent nature and non-random location in the cell nucleus, we used centromeres as proxies for higher order spatial genome organization to identify molecular determinants of global genome architecture. We tested 1064 chromatin-associated proteins in high-throughput imaging (HTI)-based CRISPR/Cas9 knockout (KO) screens in human cell lines to identify conserved molecular determinants of nuclear centromere distribution. Our data identifies proteins implicated in diverse biological functions. Furthermore, by impairing the function of several of these candidates during the cell cycle, we demonstrate that defective mitotic progression alters centromere distribution in the daughter cells. We conclude that orderly progression through mitosis shapes global 3D genome architecture.

## Results

### The spatial distribution of centromeres is cell-type specific

**Fig. 1:**
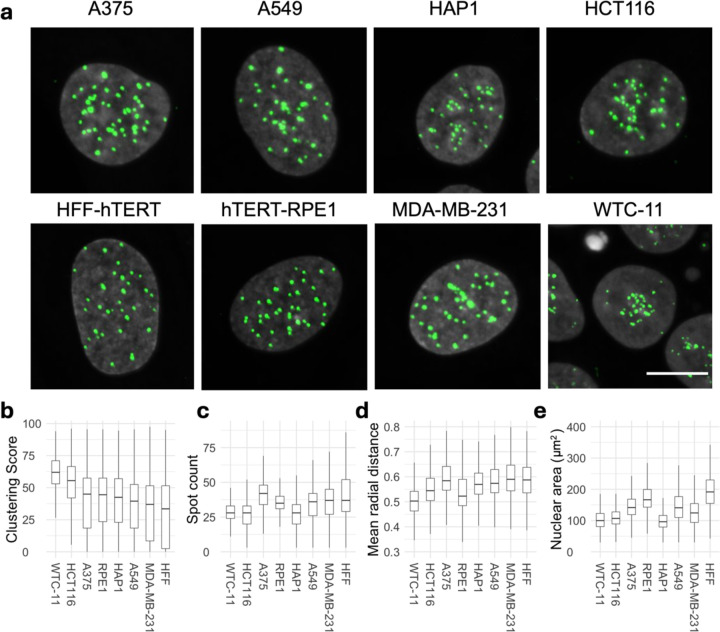
Spatial organization of centromeres is cell-type specific in human cell lines. **a**, Representative images of CENP-C (green) and DAPI (gray) stained nuclei in indicated human cell lines. Scale bar: 10 µm. **b**, **c**, Spatial organization of centromere quantified using Ripley K’s clustering score (**b**), CENP-C spot count (**c**). **d**, Nuclear area and **e**, mean radial distance in human cell lines. Statistical significance of difference between cell lines for clustering score, spot count, mean radial distance and nuclear area was tested using ANOVA (p-value or ‘Pr(>F)’ < 2e-16) following Tukey’s HSD test to compare means of all pairs of cell lines. Box plots represent the inter-quantile range (IQR) between first and third quantile (box), the median (horizontal bar), and the whiskers extend to the highest or lowest data point up to 1.5 times of IQR. Values are from one representative experiment with at least 7 technical replicates. At least 1000 cells were analyzed in each category per experiment.

We first sought to quantitatively profile the spatial distribution of centromeres in human cells (Fig. 1). We used HTI to visualize endogenous centromeres in eight cell lines from different tissues and with distinct proliferation properties, including immortalized retinal pigmented epithelium RPE1 cells, immortalized human HFF fibroblasts, the induced pluripotent stem cell (iPSC) line WTC-11, and several cancer cell lines of different origin (Data S1). Some of these cell lines contain numerical aberrations of chromosomes (Giard, Aaronson et al. 1973, Kotecki, Reddy et al. 1999), which were taken into account when setting the baseline for the quantitative analysis of centromere distribution in individual cell lines. Centromeres were visualized by indirect immunofluorescence for the integral kinetochore component CENP-C, which localizes to centromeres at all stages of the cell cycle (Hori, Amano et al. 2008, Klare, Weir et al. 2015). To confirm that CENP-C is a faithful proxy marker for centromere localization, we co-stained cells for CENP-A and CENP-C and observed that both markers co-localize throughout the cell cycle (Fig. S1a). CENP-C was used as marker for centromeres in all subsequent experiments.

We quantified the number of centromere spots per nucleus and analyzed centromere spatial distribution in the nucleus by HTI in several thousand cells per cell line by using HiTIPS, an open-source HTI analysis platform that accurately segments nuclei and centromeres in large HTI image datasets (Fig. S1b) (Keikhosravi, Almansour et al. 2024). For each cell, we measured the number of centromeres per nucleus as spot count and derived a centromere clustering score, which measures the overall distribution of centromeres in the nucleus (Fig S1c–d, also see Methods). The clustering score is a metric derived from the Ripley’s K function, which we established in pilot experiments as a robust and sensitive measure of centromere clustering (Keikhosravi, Guin et al. 2025). The clustering score quantifies deviations of the centromere distribution from uniformly distributed spots, also known as complete state of randomness (CSR), and it is robust to changes in the centromere spot number and is normalized to nuclear size (Keikhosravi, Guin et al. 2025).

We observed significant qualitative differences in centromere distribution amongst human cell lines (Fig. 1a). For example, in WTC-11 cells centromeres were strongly clustered, in line with centromere association with the nucleolus observed in other human stem cells (Wiblin, Cui et al. 2005, Rodrigues, MacQuarrie et al. 2023). In contrast, A549 basal epithelial cells derived from lung cancer and MDA-MB-231 epithelial-like breast cancer cells showed noticeably less clustering than HFF cells, which exhibited the most dispersed distribution among the cell lines tested (Fig. 1a). These visual trends were confirmed by quantitative HTI analysis using the clustering score, which was highest for WTC-11 cells and lowest for HFF cells (Fig. 1b). These differences in clustering were unrelated to spot number or to nuclear area (Fig. 1c and 1d). In addition, WTC-11 cells had the lowest and HFF cells the highest median population values for the mean normalized radial CENP-C distance, which represents the per-cell average distance of centromeres from the center of the nucleus (Fig. 1e), consistent with the differential clustering behavior in these two cell lines. Statistical analysis of variance (ANOVA) indicated that most cell lines were significantly different from each other based on centromere clustering score or spot count (Data S2), indicating cell-type specificity of centromere distribution patterns. We also noted cell-to-cell variation for all centromere distribution parameters within the population (Fig. 1b, c, e), demonstrating single-cell heterogeneity of centromere distributions as previously observed for various other features of genome organization (Finn and Misteli 2019). In conclusion, quantitative HTI analysis of centromeres localizations in thousands of single cells shows that spatial patterns of centromeres in the human cell nucleus are cell-type specific.

### Imaging-based CRISPR knockout screens identify regulators of centromere clustering

**Fig. 2:**
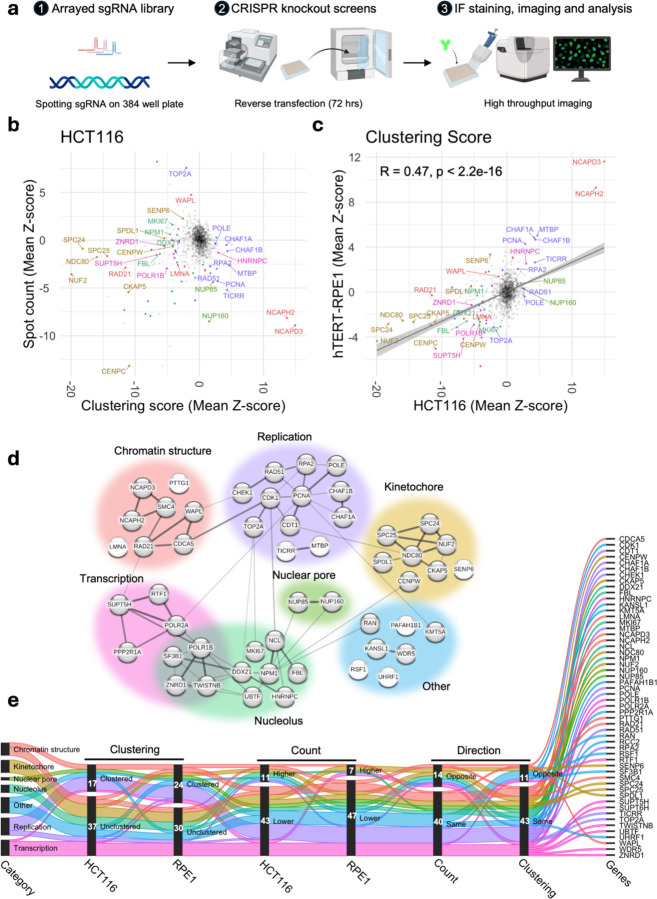
Identification of the molecular determinants of spatial centromere distribution across cell types. **a**, Schematics showing three stages of high-throughput imaging based arrayed CRISPR knockout screen employed to identify molecular determinants of spatial centromere distribution. **b**, Changes in spot count (mean Z-score of two replicates, y-axis) and clustering score (mean Z-score of two replicates, x-axis) for each of the 1064 sgRNAs. The most prominent hits were labelled and color coded as in Fig. 2d. Non-hits are colored in gray. **c**, Changes in clustering score in HCT116 (mean Z-score of two replicates, x-axis) and in RPE1 (mean Z-score of two replicates, y-axis) cells for each of the 1064 sgRNAs. Hits and non-hits are color coded and labelled as in **b**. A linear trend line (gray) was fitted to the data and Pearson’s correlation coefficient calculated is indicated at the top left corner of the plot. **d**, Network diagram with lines between 52 common hits drawn based on known physical and/or genetic interactions generated by the STRING database. Thickness of the lines indicates higher strength of data supporting the interaction. Broad categories are color coded as indicated. **e**, Changes in clustering (clustered or unclustered), count (higher or lower) and direction between two cell lines (same or opposite) for each of the common genes color coded based on their category as in Fig. 2d. Counts of genes in each subcategory are indicated. Values represent two biological replicates. Typically, 200–500 cells were analyzed for each target gene per experiment.

Having established the heterogenous and non-random nature of centromere clustering in the nucleus, we sought to identify the molecular basis for this phenomenon. To do so, we developed an arrayed HTI-based CRISPR-KO screening assay to identify regulators of the spatial distribution of centromeres (Fig. 2a). For the screens, we designed an sgRNA library targeting 1064 genes encoding nuclear proteins, enriched in structural components of the nucleus, epigenetic modifiers, and components of the genome maintenance and expression machinery (for library composition see Data S3). A non-targeting, scrambled sgRNA and sgRNAs targeting the non-expressing *OR10A5* gene were used as negative controls for sgRNA transfection and for CRISPR-induced DNA damage response (Liu, Golji et al. 2019), respectively. In addition, as a positive control for sgRNAs transfection, we used sgRNAs against the essential *PLK1* gene whose ablation results in rapid and extensive cell death (Schibler, Jevtic et al. 2023). As a positive control, we used sgRNAs targeting the condensin II complex component *NCAPH2,* whose silencing has previously been shown to induce clustering of centromeres (Hoencamp, Dudchenko et al. 2021). In light of our observation that spatial patterns of centromere distribution can be different between cell lines, we performed screens in two cell lines, HCT116 and RPE1, which represent clustered and unclustered centromere patterns, respectively (see Fig. 1a). For quantitative HTI analysis, we performed imaging-based phenotypic scoring of centromere distribution patterns using centromere spot count and the Ripley’s K-based clustering score as read-out parameters (Keikhosravi, Guin et al. 2025). All CRISPR-KO screens were performed in biological duplicates and generated data from a few hundred to over a thousand cells per replicate for each target gene (Data S4 and S5).

The results of the screens indicated consistent phenotypic separation of the positive and negative controls (Fig. S2a, b) and high reproducibility of hits in the two biological replicates for both cell lines (Fig. S2c, d). We defined hits as sgRNAs perturbations that altered either spot count or clustering Z-score by at least 2.5 units from the median phenotype of all sgRNAs included in the library (Data S3). The sgRNA KO’s that resulted in a large number of dismorphic and/or abnormally sized nuclei, as measured by nucleus compactness and/or nuclear area, were filtered out from subsequent steps of the analysis. We excluded from analysis sgRNAs which resulted in high cytotoxicity (cell number Z-score < −2.5), or which produced inconsistent results across the two biological replicates (see methods).

Following these criteria, we identified 111 genes whose CRISPR-KO altered centromere distribution in HCT116 cells (Fig. 2b, Data S4). Among these, 80% (89/111) altered the CENP-C clustering score, 41% (45/111) altered CENP-C spot counts, and 20% (23/111) altered both parameters. The majority of hits (81%; 72/89) unclustered centromeres, whereas 19% (17/89) increased clustering (Data S4). Among the 23 genes that altered both parameters, six increased the clustering score and decreased spot count, indicating higher overall clustering, whereas the opposite trend was observed for four genes, indicating overall dispersion (Data S4). The remaining genes (13/23) concomitantly decreased clustering score and spot count, suggesting global dispersion, but local clustering of centromeres into fewer but larger local clusters (Data S4). Concomitant increase of both spot count and clustering score was not observed. The effects on centromere distribution did not correlate with changes in nuclear area (Fig. S2e, f).

We similarly identified 113 hits when we performed the CRISPR-KO screen in RPE1 cells, which are characterized by a lower degree of centromere clustering than HCT116 cells (Fig. S3, Data S5). Similar to HCT116 cells, we observed a non-linear relationship of spot count and clustering score in RPE1 cells (Data S5). The majority (77%, 87/113) of identified sgRNAs altered centromere spot count, 40% altered the clustering score, while 17% altered both parameters (Fig. 2c, Data S5). When analyzed using the clustering score, the majority (58%, 26/45) of hits dispersed centromeres, whereas the rest (42%, 19/45) increased clustering (Data S5).

Select hits were orthogonally validated using siRNA knockdown with a validation rate of 90% (27/30) (Fig. S4a). Reassuringly, in line with known centromere nucleoli association (Bury, Moodie et al. 2020, van Schaik, Manzo et al. 2022), several nucleolar proteins, including NPM1, NCL and FBL, were identified as hits, confirming the validity of our screening approach. In addition, our positive control NCAPH2 represented in the library and another condensin II component NCAPD3 were strong hits in both cell lines and in all replicates of the screen. The hits were not due to secondary effects on the cell cycle, since only very few hits altered cell cycle distribution (Fig. S4b). In addition, centromere clustering levels were generally similar in G1, S and G2/M phases for most hits compared to scrambled control except for a handful of cases where the pattern changed upon knockdown of target genes (Fig. S4c).

A comparative analysis of the CRISPR-KO screen results in HCT116 and in RPE1 cells indicated that knockout of most genes similarly altered centromere distributions in both cell lines, but that the extent of change (Z-score) could vary depending on the initial state of centromere distribution (R = 0.47, p < 10^−10^, Fig. 2c). We identified 52 genes that alter centromere distribution in both cell lines (Fig. S5a). The majority of these genes altered phenotypes in the same direction for clustering score (79%, 41/52) and spot count (73%, 38/52) (Fig. 2e). Only rare examples of cell-type specific opposite effects were observed (Fig. 2e and S5b). Similarly, we identified genes that altered centromere distribution in only one cell line (Fig S5a, Data S4 and S5). Taken together, these data identify both conserved and cell-type specific regulators of centromere distribution.

To gain insights into the functions of the common hits we used STRING analysis which identifies pathways based on known physical and genetic interactions (Fig. 2d) (Szklarczyk, Nastou et al. 2025). Based on this analysis, centromere distribution modifiers were grouped into six categories: regulators of chromatin structure, kinetochore proteins, nucleolar proteins, nuclear pore complex components, replication factors and transcription-associated factors (Fig. 2d). Interestingly, while knockout of most replication- and nuclear pore- associated genes increased clustering, knockout of kinetochore components and transcription-associated factors led predominantly to centromere dispersion in both cell types (Fig. 2e and S5b). Loss of proteins implicated in chromatin structure or the nucleolus either clustered or dispersed centromeres in a gene-specific manner (Fig. 2e and Fig. S5b). These results identify major regulators of spatial centromere organization.

### Spatial re-distribution of centromeres requires cell-cycle progression

**Fig. 3:**
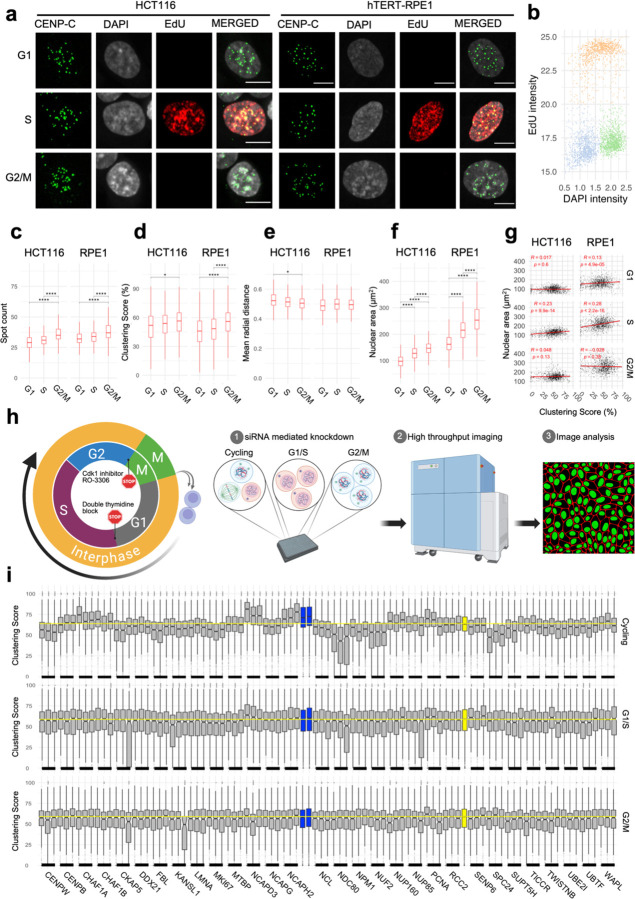
Changes in spatial organization of centromeres require progression through the cell cycle. **a**, HCT116 and RPE1 cells at G1, S and G2/M phases stained with CENP-C (green), DAPI (gray) and EdU (red). Scale bar: 10 µm. **b**, EdU intensity (y-axis) and DAPI intensity (x-axis) showing separation between G1 (brown), S (gray), and G2/M (green) sub populations in cycling HCT116 cells. Comparison of cells at G1, S or G2/M stages (x-axis) for their clustering score (c), spot count (**d**), mean radial distance (**e**), or nuclear area (**f**). Statistical significance of differences was tested by pairwise t-test with Bonferroni correction. Asterisks indicate level of significance between a given pair reflecting the corresponding p-value of that comparison. **g**, A linear regression line (red) fitted through the single cell data for nuclear area (y-axis) and clustering score (x-axis) in cells at different cell cycle phases in HCT116 and RPE1 cells. Pearson correlation coefficient and respective adjusted p-values are indicated at the top of each panel. **h,** Experimental outline to test cell cycle stage-specific effect of knocking down select hits. **i**, Effect of siRNA knockdown for a panel of genes (x-axis) using three individual siRNAs per gene in HCT116 cells that are either arrested at G/S and G2 or cycling. Two control siRNAs for siNCAPH2 are in blue and siScrambled in yellow. Mean value for siScrambled is depicted by a horizontal yellow dotted line. Statistical significance of differences was tested by performing pairwise t-tests with Bonferroni correction using siScrambled as control group. Box plots represent the inter-quantile range (IQR) between first and third quantile (box), the median (horizontal bar), and the whiskers that extend till the highest or lowest value up to 1.5 times of IQR. Values are from one representative experiment. Typically, 200 to 500 cells were analyzed in each category.

The identified regulators of centromere distribution are involved in diverse cellular functions and pathways, suggesting multi-layered control of centromere distribution. To gain mechanistic insight, we asked whether the identified regulators act at particular points in the cell cycle. To establish a baseline for analysis, we quantitated centromere distribution in G1, S and G2/M of cell-cycle staged HCT116 and RPE1 cells based on DAPI and EdU pulse-labelling as described before (Salic and Mitchison 2008, Bruhn, Kroll et al. 2014, Roukos, Pegoraro et al. 2015) (Fig. 3a, b). As expected, due to the duplication of the genome during replication, the number of detectable centromere spots increased in S phase cells (p = 0.009) and was highest in G2/M HCT116 cells (p< 10^−10^ Fig. 3c). A marginal increase in clustering score was observed in G2/M cells compared to G1 cells, (Fig. 3d; p = 0.002) while radial positioning of centromeres remained mostly unchanged except for a small increase in G1 cells (Fig. 3e; p = 0.004). A similar trend was observed in RPE1 cells for all three parameters (Fig. 3c–e). As expected, the nuclear area was significantly increased in S and G2/M in both HCT116 and RPE1 cells (Fig. 3f, p < 10^−10^). The lack of strong correlation between nuclear area and clustering score within G1, S or G2/M subpopulations (R < 0.3) indicates that increased clustering score in G2/M cells is unrelated to nuclear size increase (Fig. 3g). We conclude that, in line with observations on radial position of genomic loci (Shachar, Voss et al. 2015) and of chromosome territories (Jowhar, Gudla et al. 2018), the overall distribution of centromeres does not vary strongly within the interphase of the cell cycle.

To specifically ask whether the identified hits required progression through the cell cycle, we performed siRNA knockdown of 30 select hits in asynchronous cells or in cells that were either arrested at the G1/S boundary by standard double thymidine block (Chen and Deng 2018) or at the G2/M boundary by treatment with the CDK1 inhibitor RO-3306 as previously described (Vassilev, Tovar et al. 2006) (Fig. 3g; see Methods). While centromere distribution was altered upon knockdown of these genes in cycling cells as expected, no changes in centromere distribution were observed when knockdowns were done in G1/S or G2/M arrested cells (Fig. 3h). We conclude that while the distribution of centromeres does not vary during the cell cycle, progression through the cell cycle is required to bring about changes in centromere distribution in the absence of key regulators of centromere clustering. These results demonstrate that the identified modifiers of centromere distribution do not act in the maintenance of centromere distribution during interphase.

### Normal progression through mitosis is required for faithful interphase centromere distribution

**Fig. 4:**
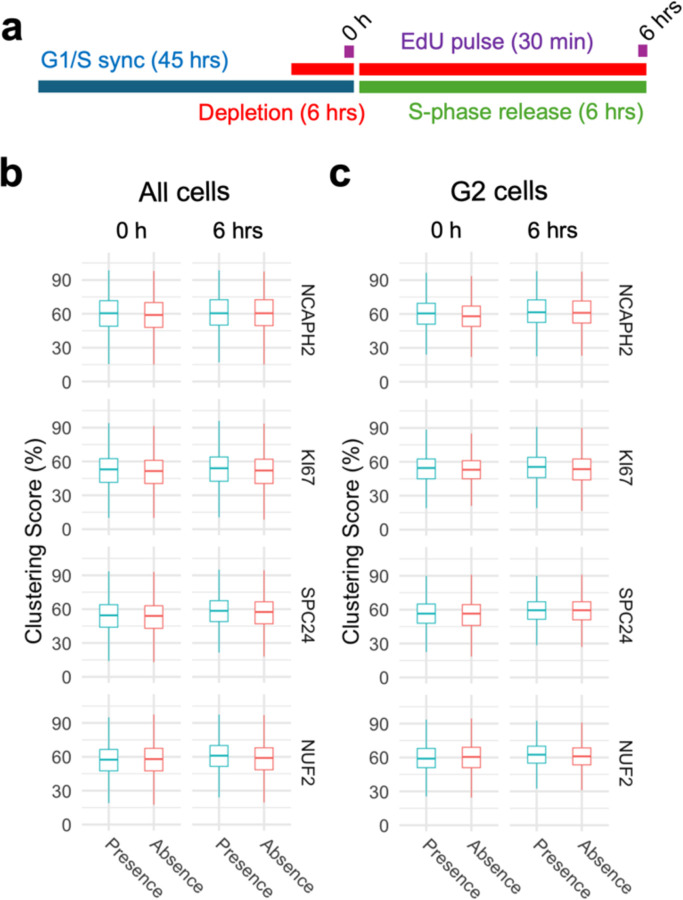
Progression through S-phase in the absence of select clustering factors does not alter interphase genome organization. **a**, Experimental outline to compare centromere distribution during progression through S-phase in the presence or absence of clustering factors. **b**, **c**, Clustering score in all cells (b) or G2/M cells (c) in the presence (blue) or absence (red) of indicated clustering factors before and after S-phase release from G1/S arrest. Pair-wise comparisons were tested with t-test with Bonferroni correction and the level of significance is indicated by asterisks if any. Pairs without significant difference are not labelled. Box plots represent the interquantile range (IQR) between first and third quantile (box), the median (horizontal bar), and the whiskers that extend till the highest or lowest value up to 1.5 times of IQR. Values are from one representative experiment with three technical replicates. Typically, 200 to 500 cells were analyzed in each category.

Having established that cell cycle progression is required for the effects of the identified centromere distribution factors, we asked at what stage of the cell cycle the centromere distribution factors act. We measured changes in clustering score before and after progressing through either S phase or mitosis in cells depleted of a given factor (Fig. 4a). We selected four proteins with distinct functions for this analysis: NCAPH2 is a component of the Condensin II complex and responsible for axial compaction of chromosomes (Shintomi and Hirano 2011, Green, Kalitsis et al. 2012, Gibcus, Samejima et al. 2018); KI67 is a well-known marker of cell proliferation that decorates nucleoli in interphase cells and coats chromosomes during mitosis (Cuylen, Blaukopf et al. 2016); SPC24 and NUF2 are kinetochore components and part of the NDC80 complex which connects the kinetochore to microtubules (McCleland, Kallio et al. 2004, Ciferri, Pasqualato et al. 2008). Auxin-inducible degron cell lines to deplete NCAPH2 and KI67 had previously been characterized (Takagi, Ono et al. 2018). In addition, we generated dTAG-SPC24 and NUF2-dTAG cell lines by CRISPR knock-in into HCT116-Cas9 parental cells (Fig. S6a, b; methods). Correct localization of dTAG-SPC24 and NUF2-dTAG was confirmed by indirect immunofluorescence staining of the tagged proteins (Fig. S6c and S6d) (McCleland, Kallio et al. 2004). Effective depletion of each factor by more than 90% as assessed by western blotting was achieved within 3 hours (Fig. S6e and f).

Using these four HCT116-based degron lines, we acutely depleted individual factors specifically in cells arrested at the G1/S or G2/M boundaries and then released the cell cycle block (see methods). First, we compared clustering scores in cells progressing through S-phase in the presence or absence of NCAPH2, KI67, SPC24, or NUF2 (Fig. 4a). Upon release from a standard double thymidine block, the majority (58–78%) of cells reached G2/M after 6 hours (Fig. S7a). Progression through S phase was equally efficient in the presence or absence of KI67, SPC24 and NUF2 (Fig. S7a). Acute NCAPH2 depletion mildly delayed S-phase progression (Figure S7a), as reported earlier (Rodemoyer, Kariyawasam et al. 2025). Regardless, no effect on clustering scores were evident as cells progressed through S-phase into G2, regardless of the presence or absence of any of these proteins (p > 0.05; Fig. 4b and c). We conclude that these centromere distribution modifiers do not act in S-phase.

**Fig. 5:**
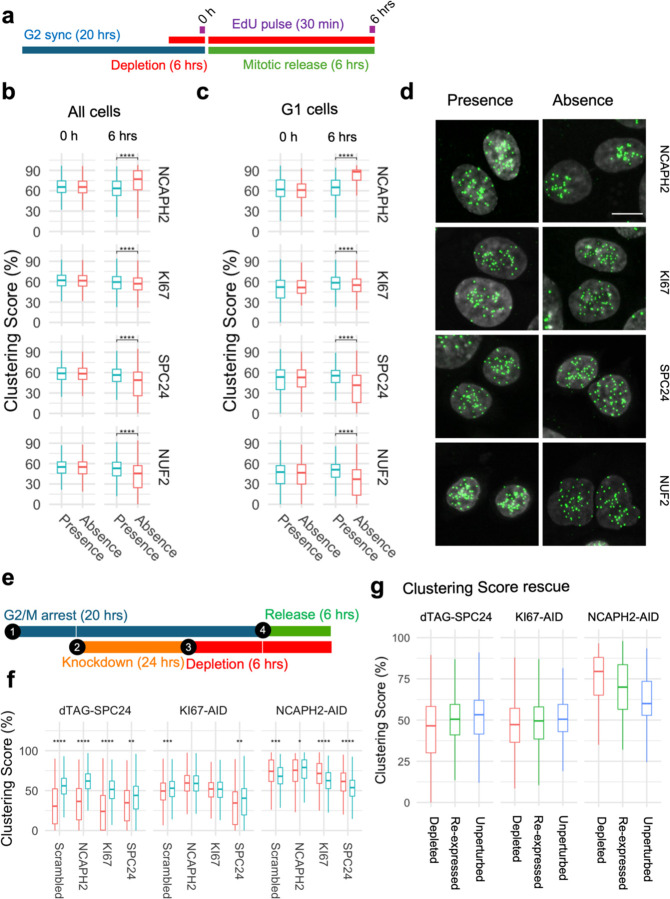
Orderly progression through mitosis is required to re-establish centromere distribution. **a**, Experimental outline to compare centromere distribution during mitotic progression in the presence or absence of clustering factors. **b**, **c**, Clustering score in all cells (b) or G1 cells (c) in the presence (blue) and absence (red) of indicated clustering factors before (0 h) and after (6 hrs) mitotic release from G2 arrest. Pair-wise comparisons were tested with t-test with Bonferroni correction and the level of significance is indicated by asterisks. Pairs without significant differences are not labelled. **d**, Representative images showing G1 nuclei stained with DAPI (gray) and CENP-C (green) in the presence or absence of indicated factors. Scale bar: 10 µm. **e**, Schematics for co-depletion of indicated factors. **f**, Clustering score (y-axis) in G1 cells after siRNA knockdown of indicated factors (x-axis) in presence (blue) or absence (red) of SPC24, KI67 or NCAPH2 as indicated. **g**, Clustering score (y-axis) in cells that were depleted (red) or depleted of indicated factors and then re-expressed (green) or remained unperturbed (blue). Box plots represent the inter-quantile range (IQR) between first and third quantile (box), the median (horizontal bar), and the whiskers that extend till the highest or lowest value up to 1.5 times of IQR. Values are from one representative experiment containing three technical replicates. Typically, 200 to 500 cells were analyzed per experiment.

Next, we tested if the loss of function of these centromere distribution modifiers during mitosis altered centromere localization in the subsequent interphase cells (Fig. 5a). HCT116 cells were arrested at the G2/M boundary by treatment for 20 hours with the CDK1 inhibitor RO-3306 as previously described (Vassilev, Tovar et al. 2006), and then released for 6 hours in the absence of each mitotic factor. The newly formed G1 cells were analyzed for centromere distribution. As expected, mitotic progression in the absence of SPC24 or NUF2 was slowed upon release from the G2/M block (McCleland, Kallio et al. 2004) with 28–35% of cells reaching G1 after 6 hours compared to 58–60% in the presence of SPC24 or NUF2 (Fig. S7b). Mitotic progression in the absence of NCAPH2 and KI67 were similar to that of control cells (Fig. S7b). While the centromere distribution phenotypes remained unaltered compared to cycling cells in the presence of these proteins, progression through a single mitosis in the absence of any of these proteins altered centromere distribution phenotypes in the subsequent G1 phase as assessed by quantitation using the Ripley’s K clustering score (Fig. 5b and c) and visual inspection (Fig. 5d). Loss of NCAPH2 had the largest effect and resulted in increased clustering of centromeres in G1 cells (Fig. 5c; p <10^−10^). Similarly, progression through mitosis in the absence of KI67 reduced clustering in the new G1 cells (p = 1.27e-08), as did loss of SPC24 (p < 10^−10^) or NUF2 (p < 10^−10^) (Fig. 5c). We conclude that NCAPH2, KI67, SPC24 and NUF2 determine centromere distribution patterns in the newly formed daughter nuclei during mitosis. The fact that loss of proteins with distinct mitotic functions perturb centromere organization in the subsequent G1 phase suggests that, rather than their specific mitotic functions, it is the orderly progression of cells through mitosis that is required to ensure the faithful maintenance of spatial centromere distribution.

Since all four factors act during mitosis but had different effects on centromere distribution, we explored co-depletion phenotypes to understand functional overlap between these factors, if any. We combined siRNA knockdown and degron-based depletion of NCAPH2, KI67 or SPC24 in pairwise combinations along with scrambled siRNAs and non-depleted cells as controls (Fig. 5e). We observed additive effects as simultaneous loss of SPC24 and KI67 further reduced the clustering score than the individual loss of either KI67 (p = 5.0640e-47) or SPC24 (p = 5.8800e-03), while the centromere unclustering upon KI67 knockdown was rescued by NCAPH2 depletion (Fig. 5f; p= 3.2280e-10). In contrast, centromeres did not cluster when *NCAPH2* was either knocked down (p= 5.9760e-56) or depleted (p= 3.2280e-10) in the absence *SPC24* (Fig. 5f) indicating that SPC24 function is dominant over NCAPH2 function. These findings indicate that these three factors act independently of each other.

We finally asked whether the aberrant altered centromere distribution in daughter cells upon depletion of mitotic factors can be reversed upon re-expression of NCAPH2, KI67 or SPC24. To test this idea, each of these factors were depleted for 6 hours in asynchronous cells following washout of degron ligands to allow re-expression of NCAPH2, KI67 or SPC24 as they progress through the cell cycle for 24 hours. Cells with and without depletion are used as controls. We observed partial rescue upon reexpression of all three factors as clustering scores partially returned towards that of the unperturbed cells (Fig. 5g).

Taken together these findings demonstrate a requirement for orderly progression through mitosis to shape the spatial distribution of centromeres and global genome organization in the subsequent interphase nuclei.

## Discussion

We identify here several cellular factors that determine the 3D positions of centromeres in the human cell nucleus, and we find that interference with orderly progression though mitosis alters centromere location in the subsequent interphase. We conclude that mitotic events shape the spatial organization of the interphase genome.

The most prominent group of centromere distribution effectors were components of the mitotic machinery, particularly multiple kinetochore proteins including all four components of the NDC80 complex (McCleland, Kallio et al. 2004) and components of the CENP-T-W-S-X complex (Nishino, Takeuchi et al. 2012). The fact that loss of multiple factors with distinct mechanisms of action, but all affecting mitosis, resulted in altered centromere distribution in the newly formed G1 cells points to a prominent role for orderly progression through mitosis as a main determinant of interphase centromere distribution, reminiscent of prior observations on lamina-associated chromatin domains (Kind, Pagie et al. 2013).

**Figure 6:**
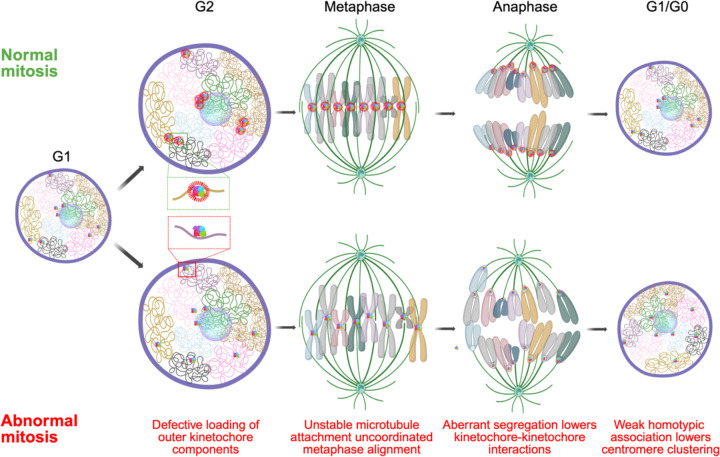
Mitotic events shape interphase genome organization. A model comparing events of normal and abnormal mitosis where defective loading of outer kinetochore (inset; green and red box) in late G2 leads to uncoordinated metaphase alignment and aberrant migration towards the spindle poles during anaphase that lowers chances of interactions between centromeres in the newly forming daughter nuclei during telophase and the lack of homotypic adhesion among centromeres contribute to further dispersion.

A likely mechanism for the observed altered arrangement of centromeres in early G1 upon interference with mitotic machinery is the aberrant alignment of chromosomes in the mitotic plate and their uncoordinated migration towards the spindle poles (Figure 6). As cells enter mitosis, the outer kinetochores assemble on the centromeres, chromosomes condense and align on the metaphase plate. This process is initiated in late G2 when the KMN (KNL1, MIS12, and NDC80) complex, including the NDC80 complex, are loaded onto the kinetochore to stabilize microtubule attachments (Gascoigne and Cheeseman 2013). Loss of NDC80 components, such as SPC24 or NUF2, weakens microtubule attachments, but does not completely disrupt chromosome segregation as has been observed for CENP-A, CENP-C and other components of inner kinetochore (Ciferri, Musacchio et al. 2007). As such, chromosomes will progress through mitosis but will be imprecisely oriented in the metaphase plate and will migrate in an un-coordinated fashion to the spindle poles, leading to their dispersal in early G1. Indeed, we find that the assembly and disassembly of the NDC80 complex correlates to lower clustering score in G1 cells compared to G2/M cells in a cycling population. This effect is further exaggerated upon knockout or depletion of multiple NDC80 complex components resulting in stronger centromere dispersion. The observed mitotic effects on interphase organization are reminiscent of recent observations on the relationship of chromosome location and missegregation defects (Klaasen, Truong et al. 2022) where single-cell observations indicated that the more peripheral a chromosome is in the interphase nucleus, the higher its chance of improper alignment in the metaphase plate and consequently being missegregated leading to aneuploidy (Klaasen, Truong et al. 2022, Vukusic and Tolic 2022). Similarly, the observed effect of NCAPH2 depletion on centromere distribution may reflect a defect in chromosome segregation. Loss of NCAPH2 has been shown to lengthen chromosomes which may facilitate homotypic centromere-centromere interactions, resulting in the observed increase in clustering of centromeres in G1 (Hoencamp, Dudchenko et al. 2021). A further contributor to the mitotic effect on interphase centromere distribution may be defects in mitotic exit as suggested by our identification of KI67 as a determinant of centromere distribution. KI67 has been localized to centromeres (van Schaik, Manzo et al. 2022) and reported to act in late telophase as a surfactant to generate mechanical forces that are required for re-establishing nuclear-cytoplasmic compartmentalization in G1 cells (Cuylen, Blaukopf et al. 2016, Hernandez-Armendariz, Sorichetti et al. 2024). Loss of KI67 may disrupt the arrangement and progression of chromosomes in late telophase leading to redistribution of centromeres in G1. Importantly, independent contributions of each factor on centromere re-distribution were reflected in our data showing additive effects during pairwise depletion of these factors.

Regardless of the precise molecular mechanisms for how mitotic progression affects the distribution of centromeres in interphase, it is likely that the location of centromeres in the nuclear space is in large parts driven by homotypic interactions. Centromeres are specialized genomic loci that are highly heterochromatic with low transcription activity (Altemose, Logsdon et al. 2022). It is well established that homotypic chromatin regions, such as heterochromatin, self-interact as is evident by the formation of the A and B chromatin compartments, which incorporate regions of similar chromatin status from distinct chromosomes (Hildebrand and Dekker 2020, Misteli 2020). A homotypic self-organization model for centromeres is in line with the presence of chromocenters in mouse, fly and plant cells, which represent clusters of centromeres from multiple chromosomes forming large heterochromatin blocks (Sullivan and Karpen 2004, Probst and Almouzni 2011, Jagannathan, Cummings et al. 2019), but are largely absent in humans indicating factors implicated in chromocenter maintenance are probably not required for spatial centromere organization in humans. Indeed, we find that knockout of *HMGA1,* whose gene product stabilizes chromocenters in mouse (Jagannathan, Cummings et al. 2018), or other HMG genes, did not affect the spatial distribution of centromeres in human cells, suggesting species-specificity of some determinants of genome organization. A hetero-chromatin-driven homotypic interaction model also explains the prominent association of centromeres with the nucleolus in human stem cells, which are largely devoid of nuclear heterochromatin blocks (Meshorer and Misteli 2006), making the nucleolus the most prominent high-affinity binding site for centromeres in the nucleus. Our model is also in line with the long-standing observation that following mitosis, chromosome unfolding leads to the re-establishment of the chromatin landscape of interphase nuclei (Belmont and Bruce 1994).

In conclusion, our study reveals several major regulators of centromere distribution in human cell types and demonstrates that mitotic events shape interphase spatial genome organization.

## Supplementary Material

Supplement 1
