# Supplementary material for: Orderly mitosis shapes interphase genome architecture
Source: bioRxiv. 2025 Jul 10:2025.06.03.657645. Originally published 2025 Jun 3. Preprint. [Version 2] doi: 10.1101/2025.06.03.657645 (PMC12157558; doi:10.1101/2025.06.03.657645)
Supplement: Supplement 1 [file media-1.pdf]

**Supplementary materials for**

# Orderly mitosis shapes interphase genome architecture

Krishnendu Guin[1], Adib Keikhosravi[2], Raj Chari[3], Gianluca Pegoraro[2], Tom Misteli[1]

[1]Cell Biology of Genomes, National Cancer Institute, NIH, Bethesda, MD 20892, USA

[2]High Throughput Imaging Facility (HiTIF), National Cancer Institute, NIH, Bethesda, MD 20892, USA

[3]Genome Modification Core (GMC), Frederick National Lab for Cancer Research, Frederick, MD 21702, USA

Correspondence to: mistelit@mail.nih.gov

## The PDF file includes:

Figs. S1 to S7

## Other Supplementary Materials for this manuscript include the following:

Data S1 to S6

## Supplementary figures

**Fig. S1: Quantification of centromere clustering using CENP-A and CENP-C as centromere markers.**

**a,** Co-staining of HCT116 cells with CENP-A (red), CENP-C (green) and DAPI (gray). Scale bar: 10 μm **b**, Representative image showing segmentation of DAPI stained nuclei (gray) and CENP-C-stained centromere spots (green) in high-throughput imaging data using HiTIPS. Red lines around the DAPI stained nuclei indicate nuclear segmentation, the green circles around CENP-C spots indicate segmentation of centromeres. Zoomed images of the same nucleus are shown with yellow and pink borders indicate before and after spot segmentation was applied. Scale bar: 10 μm. **c**, Quantification of spot count and **d**, clustering score using CENP-A (red) and CENP-C (blue) as centromere markers in HCT116 cells. Values are from two replicates, with at least 2000 cells analyzed for each experimental condition.

**Fig. S2. CRISPR knockout screens for centromere distribution phenotypes in HCT116 cells are reproducible.**

**a**, Mean and standard deviation for phenotypic separation between control sgRNAs with individual data points representing mean value per well for number of spots per nucleus and **b**, clustering score in two biological replicates. **c**, scatter plot showing changes in clustering score or **d**, spot count for replicate 1 (x-axis) and replicate 2 (y-axis) in HCT116 cells for each of the 1068 sgRNAs. A linear regression line (gray) was fitted to the data and Pearson's correlation coefficient calculated is indicated at the top left corner of the plot. **e**, scatter plot and linear regression line correlating changes in clustering score (y-axis) and **f,** spot count (y-axis) with nuclear area (x-axis). Pearson's correlation coefficients and corresponding p-values are indicated at the top left corner. Values are from 2 biological replicates. Typically, 200 to 500 cells were imaged for each target gene per experiment.

**Fig. S3: Identification of the molecular determinants of spatial centromere distribution in RPE1 cells.**

Changes in spot count (mean Z-score of two replicates, y-axis) and clustering score (mean Z-score of two replicates, x-axis) for each of the 1068 sgRNAs. The most prominent hits were labelled and color coded as in Fig. 2d. Non-hits are colored in gray. Values are from two biological replicates. Typically, 200 to 500 cells were analyzed for each target gene per experiment.

**Figure S4: Cell cycle analysis of clustering factor knockdown and their effect on clustering score.**

**a**, Clustering scores of targets (x-axis) after siRNA knockdown in HCT116 cells. Two control siRNAs for siNCAPH2 are in blue and siScrambled is shown in yellow. Upon siRNA knockdown, the clustering score or spot count for the targets (x-axis) labelled in green change in the same direction as in the CRISPR-KO screens and are compared to the mean value for siScrambled as depicted by a horizontal yellow dotted line. Targets in gray disagree with either clustering score or spot count or both parameters compared to data in CRISPR-KO screens. Three separate siRNA were used per target. **b**, Fraction of cells in each cell cycle stage (x-axis) after knockdown of select targets as indicated (y-axis). Individual sub-populations of the cell cycle are color-coded as identified using DAPI and EdU fluorescence intensity measurement, and their percentages are indicated. **c**, Clustering score (y-axis) for select targets (x-axis) at G1 (brown), S (gray) and G2/M (green) stages. Values are from one representative experiment. Typically, 200 to 500 cells were analyzed per gene per experiment.

**Fig. S5: Comparative analysis of cell lines confirms common molecular determinants of spatial centromere distribution.**

**a,** 52 genes (black mesh) that are hits in both HCT116 and RPE1 cells. A total of 113 hits were selected in RPE1 cells for either clustering score (pink, 45) or spot count (blue, 87) and 111 hits in HCT116 cells for either clustering score (gold, 89) or spot count (black, 45). White non-shaded areas indicate unique hits in HCT116 (51) and RPE1 (53) cells. Values are from one representative experiment. Typically, 200 to 500 cells were analyzed for each target gene per experiment. **b**, Z-scores (x-axis) of spot count and clustering

score for the 52 common hits (y-axis) in HCT116 and RPE1 cells. Genes are color coded based on their category as indicated in Fig. 2e.

**Fig. S6: Construction and characterization of FLAG-dTAG-SPC24 and NUF2-dTAG-FLAG cell lines.**

**a**, **b**, CRISPR knock-in strategy for homozygous tagging of SPC24 (**a**) and NUF2 (**b**) with the dTAG-FLAG epitope. Horizontal black arrows indicate positions of primers used for PCR confirmation of the tagged allele. **c**, **d**, Representative images of knock-in cell lines expressing FLAG-dTAG-SPC24 (**c**), and NUF2-dTAG-FLAG (**d**) stained with DAPI (gray), CENP-C (green) and FLAG (red). **e**, **f**, Westen blot images showing levels of dTAG-SPC24 (**e**), and NUF2-dTAG (**f**) at indicated times after incubation with dTAG ligands and the relative ratios of dTAG-SPC24 or NUF2-dTAG to tubulin control at the beginning of depletion (0 hr) are indicated below.

**Fig. S7: Quantification of cell cycle stages during G1 and mitotic release experiment.**

**a**, Fraction of G1 (blue), S (green) and G2/M (orange) cells were quantified before (0h) and after (6h) release from double thymidine block in the presence or absence of indicated clustering factors. **b**, Fraction of G1 (blue), S (green) and G2/M (orange) cells were quantified before (0h) and after (6h) release from G2/M block in the presence or absence of indicated clustering factors. Values are from one representative experiment containing three technical replicates. Typically, 200 to 500 cells were analyzed per sample.

Fig. S1

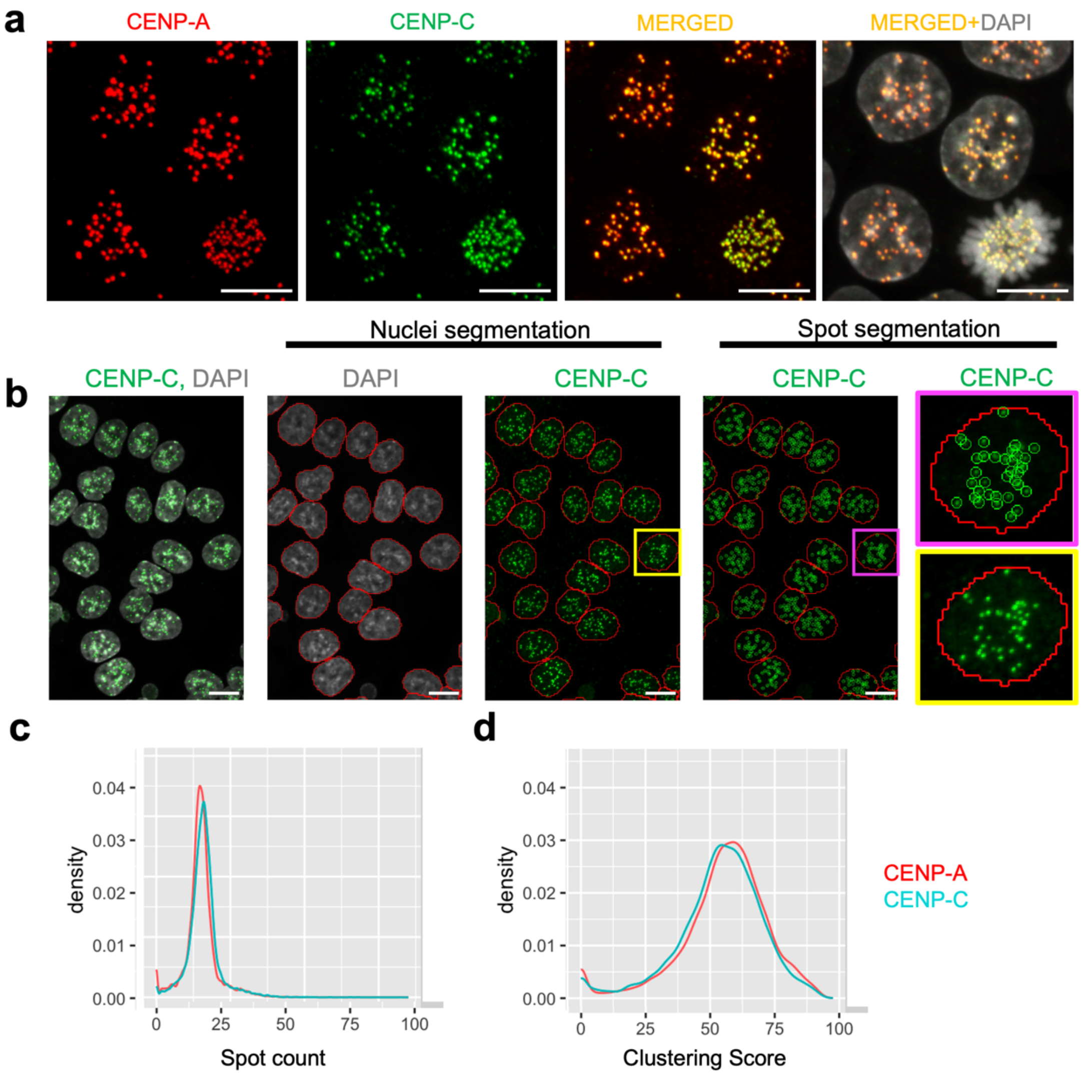

a
CENP-A
CENP-C
MERGED
MERGED+DAPI
Nuclei segmentation
Spot segmentation
b
CENP-C, DAPI
DAPI
CENP-C
CENP-C
CENP-C
c
0.04
0.03
0.02
0.01
0.00
density
0
25
50
75
100
Spot count
d
0.04
0.03
0.02
0.01
0.00
density
0
25
50
75
100
Clustering Score
CENP-A
CENP-C

Fig. S2

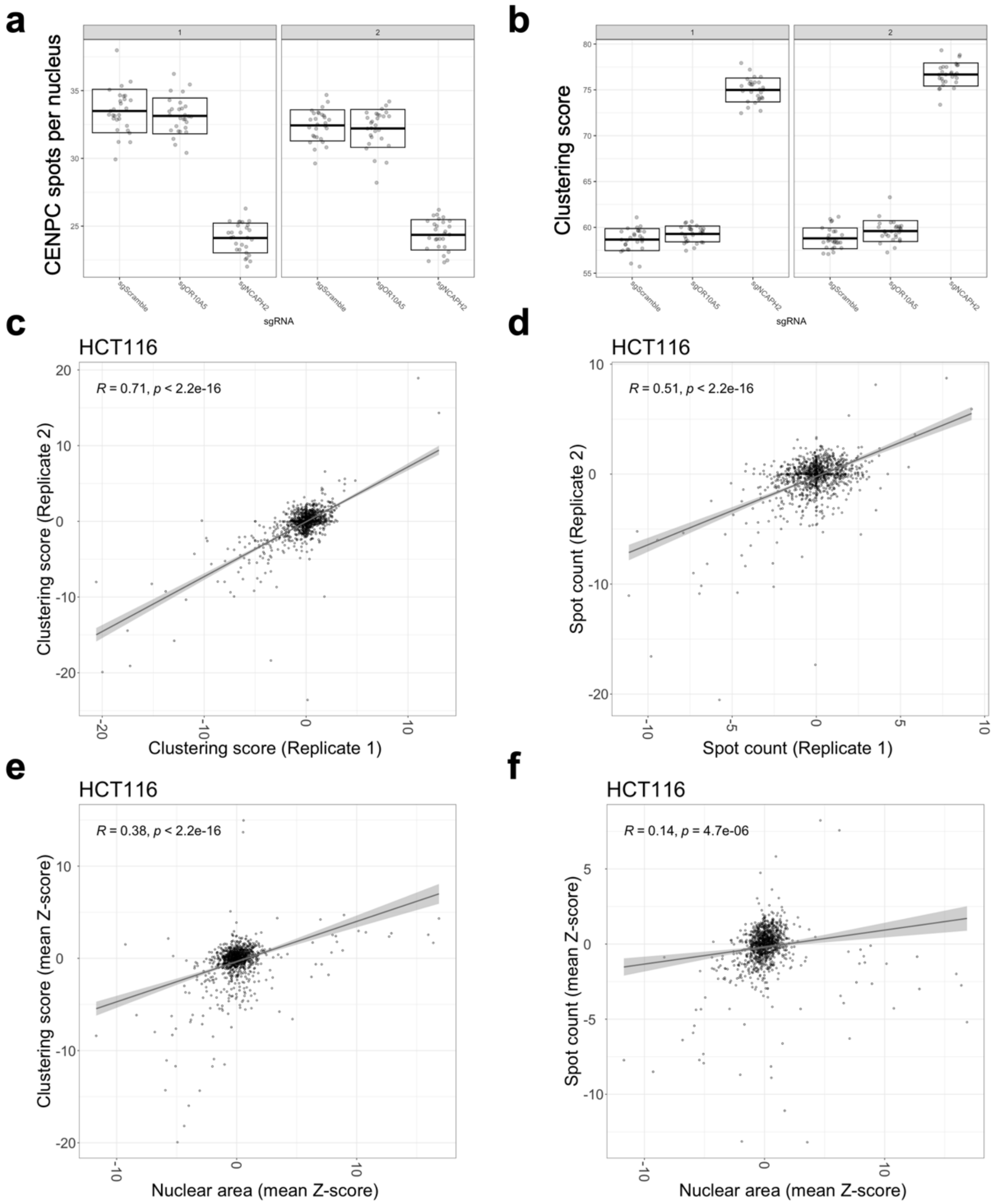
a
CENPC spots per nucleus
sgScramble
sgOR10A5
sgNCAPH2
sgRNA
b
Clustering score
c
HCT116
R = 0.71, p < 2.2e-16
Clustering score (Replicate 2)
Clustering score (Replicate 1)
d
HCT116
R = 0.51, p < 2.2e-16
Spot count (Replicate 2)
Spot count (Replicate 1)
e
HCT116
R = 0.38, p < 2.2e-16
Clustering score (mean Z-score)
Nuclear area (mean Z-score)
f
HCT116
R = 0.14, p = 4.7e-06
Spot count (mean Z-score)
Nuclear area (mean Z-score)

Fig. S3

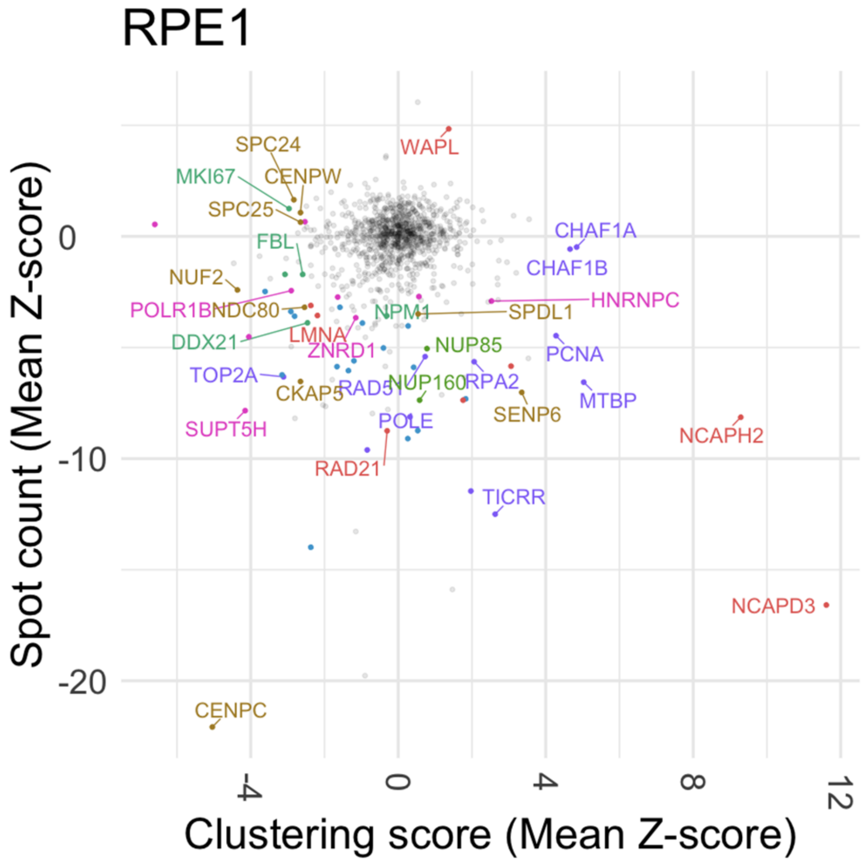
RPE1
Spot count (Mean Z-score)
Clustering score (Mean Z-score)
0
-10
-20
-4
0
4
8
12
WAPL
SPC24
MKI67
CENPW
SPC25
CHAF1A
FBL
CHAF1B
NUF2
HNRNPC
POLR1B
NDC80
NPM1
SPDL1
DDX21
LMNA
NUP85
ZNRD1
PCNA
TOP2A
NUP160
RPA2
RAD51
CKAP5
MTBP
SENP6
SUPT5H
POLE
NCAPH2
RAD21
TICRR
NCAPD3
CENPC

# Fig. S4

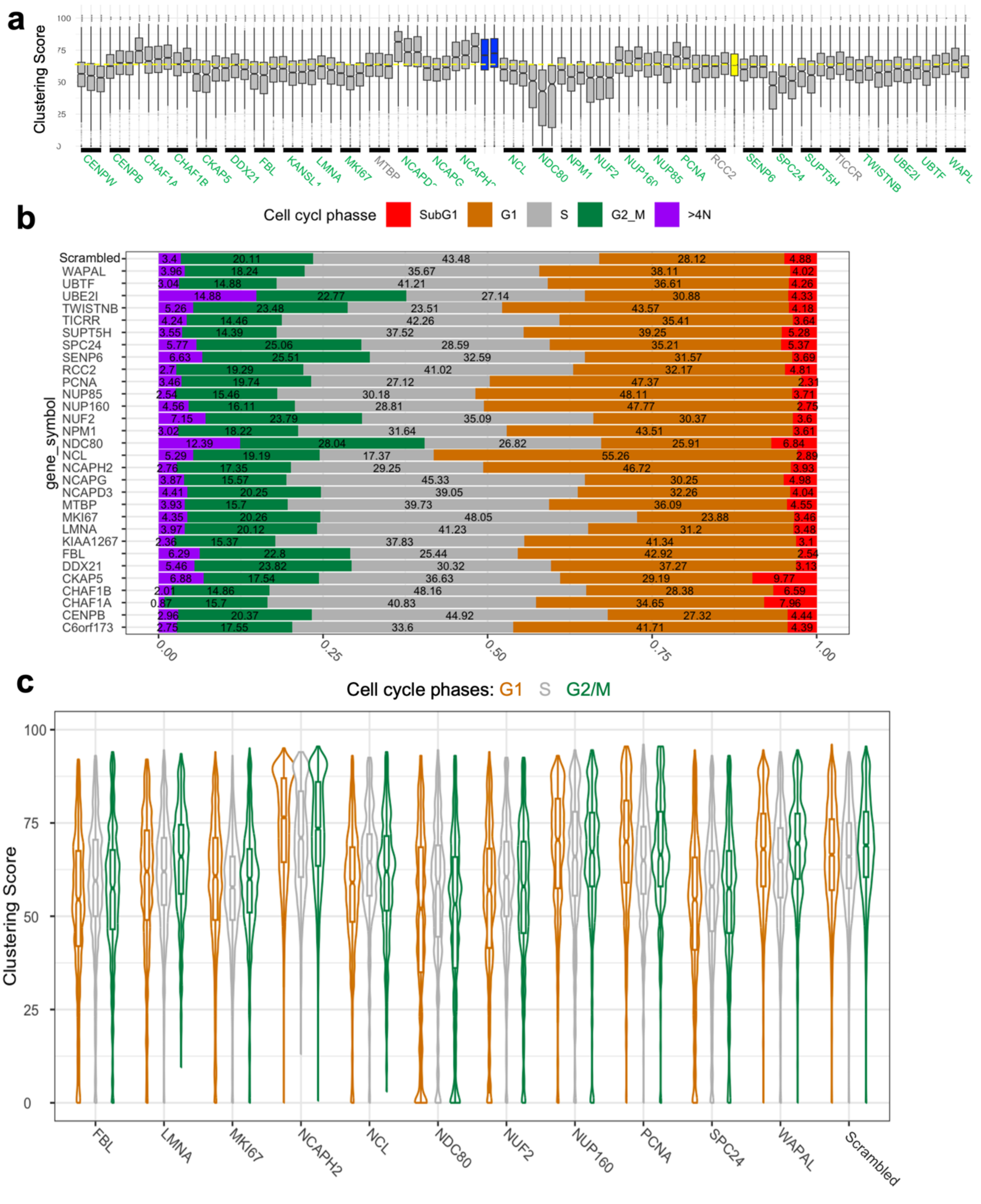
a
Clustering Score
CENPW
CENPB
CHAF1A
CHAF1B
CKAP5
DDX21
FBL
LMNA
MKI67
MTBP
NCL
NDC80
NPM1
NUF2
NUP160
NUP85
PCNA
RCC2
SENP6
SPC24
SUPT5H
TICCR
TWISTNB
UBE2I
UBTF
b
Cell cycl phasse
SubG1
G1
S
G2_M
>4N
gene_symbol
Scrambled
WAPAL
UBTF
UBE2I
TWISTNB
TICRR
SUPT5H
SPC24
SENP6
RCC2
PCNA
NUP85
NUP160
NUF2
NPM1
NDC80
NCL
NCAPH2
NCAPG
NCAPD3
MTBP
MKI67
LMNA
KIAA1267
FBL
DDX21
CKAP5
CHAF1B
CHAF1A
CENPB
C6orf173
0.00
0.25
0.50
0.75
1.00
c
Cell cycle phases: G1 S G2/M
Clustering Score
100
75
50
25
0
FBL
LMNA
MKI67
NCAPH2
NCL
NDC80
NUF2
NUP160
PCNA
SPC24
WAPAL
Scrambled

Fig. S5

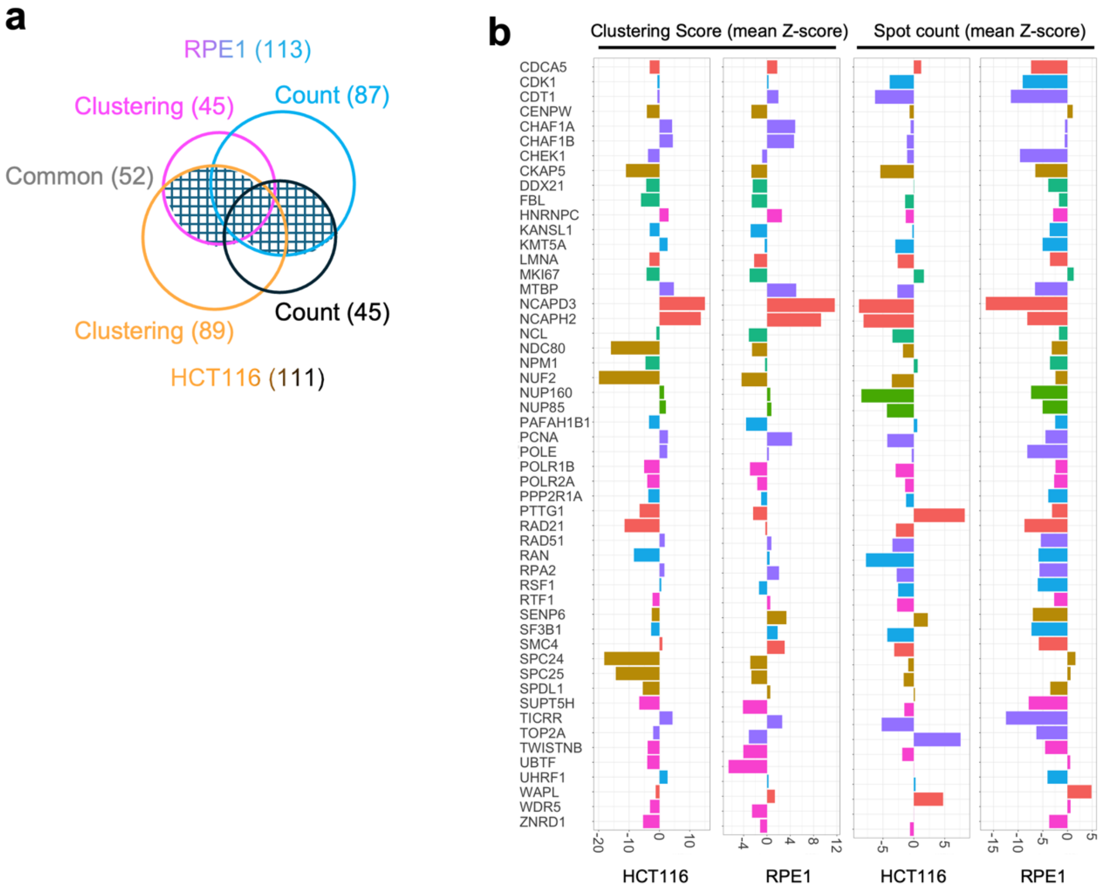
a
RPE1 (113)
Clustering (45)
Count (87)
Common (52)
Count (45)
Clustering (89)
HCT116 (111)
b
Clustering Score (mean Z-score)
Spot count (mean Z-score)
CDCA5
CDK1
CDT1
CENPW
CHAF1A
CHAF1B
CHEK1
CKAP5
DDX21
FBL
HNRNPC
KANSL1
KMT5A
LMNA
MKI67
MTBP
NCAPD3
NCAPH2
NCL
NDC80
NPM1
NUF2
NUP160
NUP85
PAFAH1B1
PCNA
POLE
POLR1B
POLR2A
PPP2R1A
PTTG1
RAD21
RAD51
RAN
RPA2
RSF1
RTF1
SENP6
SF3B1
SMC4
SPC24
SPC25
SPDL1
SUPT5H
TICRR
TOP2A
TWISTNB
UBTF
UHRF1
WAPL
WDR5
ZNRD1
-20 -10 0 10
-4 0 4 8 12
-5 0 5
-15 -10 -5 0 5
HCT116
RPE1
HCT116
RPE1

Fig. S6

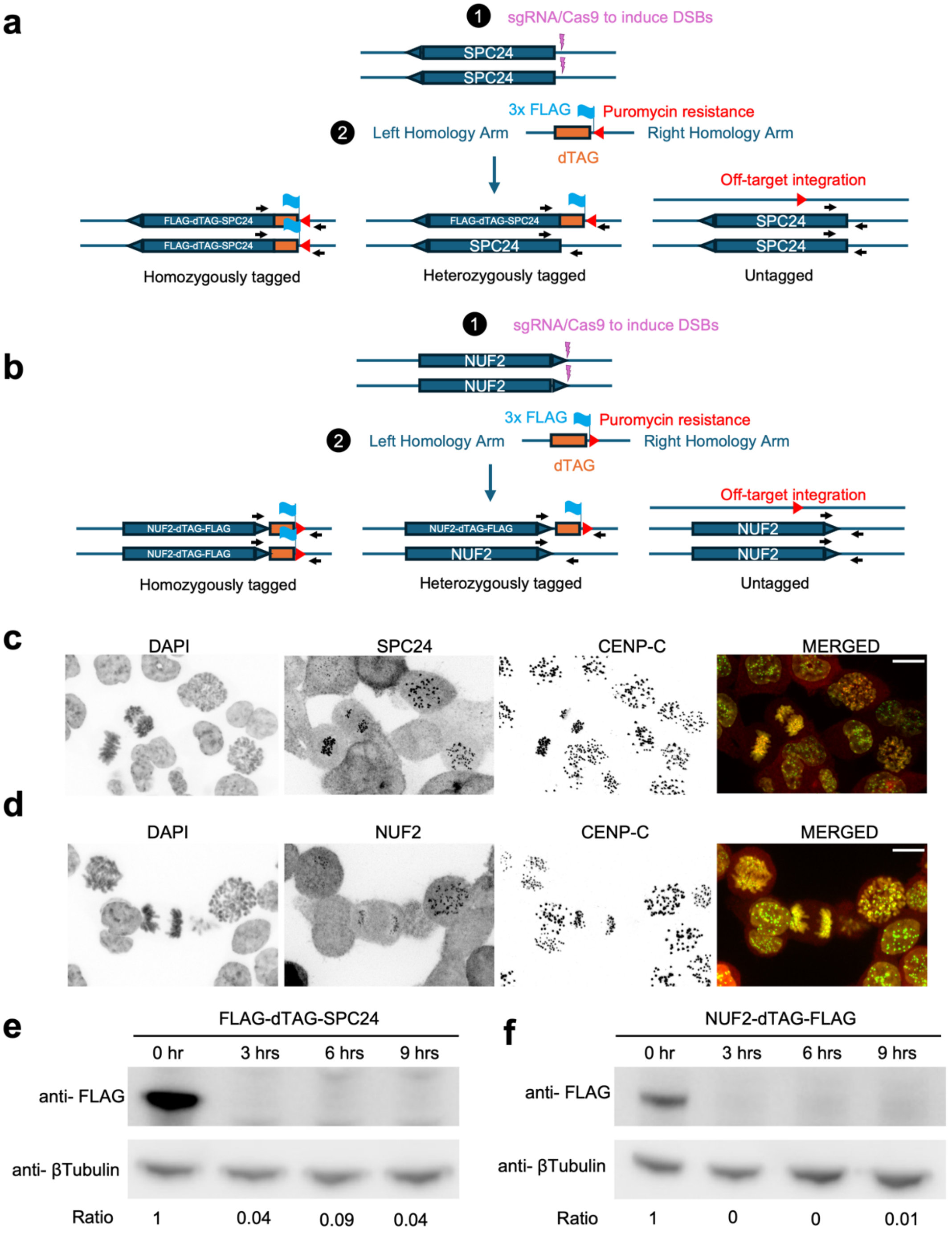
a
1
sgRNA/Cas9 to induce DSBs
SPC24
SPC24
3x FLAG
Puromycin resistance
2
Left Homology Arm
Right Homology Arm
dTAG
Off-target integration
FLAG-dTAG-SPC24
FLAG-dTAG-SPC24
FLAG-dTAG-SPC24
SPC24
SPC24
SPC24
Homozygously tagged
Heterozygously tagged
Untagged
b
1
sgRNA/Cas9 to induce DSBs
NUF2
NUF2
3x FLAG
Puromycin resistance
2
Left Homology Arm
Right Homology Arm
dTAG
Off-target integration
NUF2-dTAG-FLAG
NUF2-dTAG-FLAG
NUF2-dTAG-FLAG
NUF2
NUF2
NUF2
Homozygously tagged
Heterozygously tagged
Untagged
c
DAPI
SPC24
CENP-C
MERGED
d
DAPI
NUF2
CENP-C
MERGED
e
FLAG-dTAG-SPC24
0 hr
3 hrs
6 hrs
9 hrs
anti- FLAG
anti- βTubulin
Ratio
1
0.04
0.09
0.04
f
NUF2-dTAG-FLAG
0 hr
3 hrs
6 hrs
9 hrs
anti- FLAG
anti- βTubulin
Ratio
1
0
0
0.01

Fig. S7

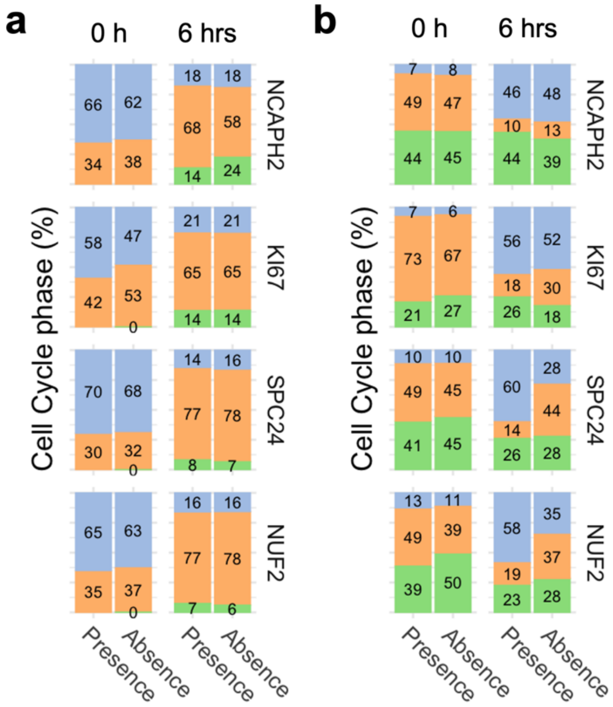
a
0 h
6 hrs
b
0 h
6 hrs
Cell Cycle phase (%)
NCAPH2
KI67
SPC24
NUF2
Presence
Absence
Presence
Absence
66
62
34
38
18
18
68
58
14
24
58
47
42
53
0
21
21
65
65
14
14
70
68
30
32
0
14
16
77
78
8
7
65
63
35
37
0
16
16
77
78
7
6
7
8
49
47
44
45
46
48
10
13
44
39
7
6
73
67
21
27
56
52
18
30
26
18
10
10
49
45
41
45
60
28
14
44
26
28
13
11
49
39
39
50
58
35
19
37
23
28
