# Supplementary material for: Orderly mitosis shapes interphase genome architecture
Source: bioRxiv. 2025 Jun 3:2025.06.03.657645. Preprint. [Version 1] doi: 10.1101/2025.06.03.657645 (PMC12157558; doi:10.1101/2025.06.03.657645)
Supplement: Supplementary file 1 [file NIHPP2025.06.03.657645v1-supplement-1.pdf]

bioRxiv preprint doi: https://doi.org/10.1101/2025.06.03.657645; this version posted June 3, 2025. The copyright holder for this preprint (which was not certified by peer review) is the author/funder. This article is a US Government work. It is not subject to copyright under 17 USC 105 and is also made available for use under a CC0 license.

Supplementary materials for

Orderly mitosis shapes interphase genome architecture

Krishnendu Guin[1], Adib Keikhosravi[2], Gianluca Pegoraro[2], Raj Chari[3], Tom Misteli[1]

[1]Cell Biology of Genomes, National Cancer Institute, NIH, Bethesda, MD 20892, USA

[2]High Throughput Imaging Facility (HiTIF), National Cancer Institute, NIH, Bethesda, MD 20892, USA

[3]Genome Modification Core (GMC), Frederick National Lab for Cancer Research, Frederick, MD 21702, USA

Correspondence to: mistelit@mail.nih.gov

The PDF file includes:

Materials and Methods
Figs. S1 to S7
References

Other Supplementary Materials for this manuscript include the following:

Data S1 to S6

bioRxiv preprint doi: https://doi.org/10.1101/2025.06.03.657645; this version posted June 3, 2025. The copyright holder for this preprint (which was not certified by peer review) is the author/funder. This article is a US Government work. It is not subject to copyright under 17 USC 105 and is also made available for use under a CC0 license.

Materials and Methods

Cell Culture

All cell lines used in this study are grown in a humidified 37°C incubator in presence of 5% CO_2. Sources, composition of growth media, relevant references of the respective cell culture protocols are in **Data S1**. Cells were fixed with 2% paraformaldehyde (PFA, Electron Microscopy Sciences, Cat. No. 15710) solution in media by adding one equal volume of 4% PFA solution in PBS to the cell growth medium in each well for 15 minutes at room temperature. To activate protein degradation 500 nM auxin (Millipore Sigma, Cat. No. I3750-25G-A) or 1 µM dTAG13 (Tocris Bioscience, Cat. No. 6605) or dTAG^{V}-1 (Tocris Bioscience, Cat. No. 6914) ligand was used.

Cell cycle synchronization

Cells were synchronized at G1/S boundary by double thymidine block as described (Chen and Deng 2018). Briefly, cells were seeded at 20-30% confluency and grown for 24 hours following 18 hours of growth in media containing 2 mM thymidine (Sigma, Cat. No. T9250-5G). Cells were then washed with PBS and grown in fresh media for 9 hours. The growth media was changed with fresh media containing 2 mM thymidine. Cells were synchronized at the G2/M stage by treating cells with 9 µM RO-3306 (Millipore Sigma, Cat No. 217721-2MG) for 20 hours as described (Vassilev, Tovar et al. 2006). Cells were once washed in 300 µL fresh growth media and replenished with fresh growth media to release from the G1/S or G2/M block.

Immunofluorescence staining

Indirect immunofluorescence (IF) staining was performed as previously described (Keikhosravi, Almansour et al. 2024, Keikhosravi, Guin et al. 2025). Briefly, fixed cells grown on a 96 well plate (Revvity, Cat. No. 6055300) or 384 well plate (CellVis, Cat. No. P384-1.5H-N) were washed with PBS and then permeabilized with 0.1% Triton X-100 (Sigma, Cat. No. T9284-500ML) solution in PBS for 15 minutes and again washed with PBS. These cells were blocked by incubating with 5% BSA (Millipore Sigma, Cat. No.

bioRxiv preprint doi: https://doi.org/10.1101/2025.06.03.657645; this version posted June 3, 2025. The copyright holder for this preprint (which was not certified by peer review) is the author/funder. This article is a US Government work. It is not subject to copyright under 17 USC 105 and is also made available for use under a CC0 license.

A3294-100G) solution in PBST (0.05% Tween-20 in PBS) for 15 min at room temperature. Next, cells were incubated with appropriate primary antibody dilution prepared in blocking solution (5% BSA in PBST) for 1 hour at room temperature and then washed with PBS three times. Next cells were incubated for 1 hour with fluorescently labelled secondary antibody solution prepared in blocking solution at room temperature and then washed with PBS three times. DAPI (4′,6-diamidino-2-phenylindole) (Thermo Fisher Scientific, Cat. No. 62248) staining was performed by adding 5 µg/mL DAPI solution prepared in 1x PBS to the wells. Anti-CENP-C (MBL Biosciences, Cat. No. PD030) and anti-CENP-A (Abcam, Cat. No. AB13939) antibodies were diluted 1:1000 in blocking buffer (5% BSA solution prepared in PBST) and used for IF staining. In experiments where cells were EdU labelled, CENP-C primary antibody was directly conjugated with fluorophores using Mix-n-Stain™ CF® Dye Antibody Labeling Kit (Biotium, Cat. No. 922235). Anti-FLAG monoclonal antibody (Sigma, cat. No. F3165250) was diluted 1:250 in blocking buffer and used for IF staining.

CRISPR-KO Library design

A custom arrayed synthetic sgRNA library targeting 1064 genes associated with chromatin biology and nuclear architecture was sourced from Synthego (Cat # SO17105 and 8311960), delivered lyophilized in 96-well plates, resuspended in RNAse free ddH_2O, and reformatted in 384-well plate format using the PerkinElmer Janus and the Beckman Coulter ECHO525 liquid handlers at a final of 0.25 pmoles/µL. Each gene was targeted in the same well by 3 pooled sgRNA oligos that included the Synthego modified EZ Scaffold. The list of genes and sgRNA targeting sequences in the library is included in Data S3.

CRISPR knockout screens

For reverse transfection of sgRNA oligos in 384-well format, 325 nL of library sgRNA 0.25 pmoles/µL were spotted in each empty well (0.08 pmoles/well) of an imaging plate (CellVis, Cat. No. P384-1.5H-N) using an ECHO525 acoustic liquid handler. As controls, and in each plate, we also spotted 7 wells each of non-targeting scrambled control sgRNA (Synthego, Cat. No. 063-1010-000-000), sgRNAs targeting each of *PLK1, OR10A5*, and

bioRxiv preprint doi: https://doi.org/10.1101/2025.06.03.657645; this version posted June 3, 2025. The copyright holder for this preprint (which was not certified by peer review) is the author/funder. This article is a US Government work. It is not subject to copyright under 17 USC 105 and is also made available for use under a CC0 license.

NCAPH2. Three sgRNAs pooled together in gene knockout kits to target *PLK1*, *OR10A5* and *NCAPH2* were obtained from Synthego (Cat. No. GKO-HS1-000-0-1.5n-0-0). The control sgRNAs had the same chemistry and were spotted in the same quantities as the as the sgRNAs in the library. Spotted plates were dried at RT under a laminar flow cell culture hood, sealed, and stored at -30°C until the day of the reverse transfection.

The day of the transfection, the spotted sgRNA imaging plates were thawed and equilibrated at RT and then spun at 1400 rpm. The seal was removed, and 20 µL of prewarmed serum-free Optimem media (Thermo Fisher Scientific, Cat. No. 31985070)) was dispensed into each well for the imaging plate using a Thermo Fisher Multidrop dispenser. The ECHO525 was then used to dispense required amount of Lipofectamine RNAi MAX (Thermo Fisher Scientific, Cat. No. 13778075). The plates were then incubated at room temperature for 30 minutes to allow RNA-lipofectamine complexes to form. Next, a cell suspension prepared in prewarmed Optimem media (Thermo Fisher Scientific, Cat. No. 31985070) containing 20% FBS was dispensed into each well using the Multidrop for a total volume of 40 µl and an effective final sgRNA concentration of 2 nM. Plates were incubated at room temperature inside a laminar airflow hood for 30 minutes before they were transferred into cell culture incubator and allowed to grow at for 72 hours. CRISPR-KO screens were performed each in 2 biological replicates on different days.

Imaging

384 well plates containing fixed cells were then stained for CENP-C and DAPI and imaged using a Yokogawa CV8000 spinning disk confocal microscope. Imaging parameters were as described before (Keikhosravi, Almansour et al. 2024). Briefly, Immunofluorescence images were collected on a multi-laser platform equipped with 405 nm (DAPI), 488 nm (for green fluorophores), 561 nm (for red fluorophores), and 640 nm (for far-red fluorophores) excitation lines that were combined through a 405/488/561/640 nm quad-band dichroic. Fluorescence was captured through a 60× water-immersion objective (NA = 1.2) and routed to either a 445/45 nm band-pass filter for DAPI or a 525/50 nm band-pass filter for green fluorophores, or 600/37 nm band-pass filter for red fluorophores or

bioRxiv preprint doi: https://doi.org/10.1101/2025.06.03.657645; this version posted June 3, 2025. The copyright holder for this preprint (which was not certified by peer review) is the author/funder. This article is a US Government work. It is not subject to copyright under 17 USC 105 and is also made available for use under a CC0 license.

676/29 nm band-pass filter for far-red fluorophores. A 16-bit sCMOS detector (2048 × 2048 pixels, 1 × 1 binning; effective pixel size = 0.108 μm) recorded Z-stacks with 1 μm steps, while real-time maximal projection was applied. Variable number of fields ranging from 9-22 were imaged per well in different imaging experiments to acquire sufficient number of cell images.

Image analysis

Image analysis was performed as described (Keikhosravi, Guin et al. 2025). Raw image stacks were processed with HiTIPS, our previously described high-content analysis pipeline for fixed- and live-cell assay (Keikhosravi, Almansour et al. 2024). Max-projected DAPI channels provided nuclear masks, whereas CENP-A or CENP-C projections served for centromere spot localization (see **Fig. S1B**). Analysis settings in HiTIPS were adjusted to the typical nuclear diameter and the intensity/size characteristics of centromere foci. Nuclear segmentation employed the GPU-accelerated CellPose algorithm (Pachitariu and Stringer 2022), and centromere detection used a Laplacian-of-Gaussian approach. Final spot coordinates were defined as the centroid of each segmented focus. Average nuclear fluorescence intensity for the DAPI (405 nm) and EdU (640 nm) channels were measured at the single cell level. Clustering scores were calculated using a metric derived from Ripley's K function as described (Keikhosravi, Guin et al. 2025).

Identification of screen hits

Single cell data obtained from HiTIPS (Nucleus area, Number of CENP-C Spots per Cell, Ripley's K clustering score, mean normalized CENP-C Spot Radial distance) were averaged on a per well level. Per well average values and nuclei counts was then used as an input for the screen statistical analysis using R 4.3.3 [(R Core Team (2024). _R: A Language and Environment for Statistical Computing_. R Foundation for Statistical Computing, Vienna, Austria. <https://www.R-project.org/>] and the cellHTS2 package (Boutros, Bras et al. 2006). Briefly, per well raw measurement were normalized on a per plate basis using the median value of the sgRNA library treatments and the B-score method. All the per plate normalized values in different library plates originated from a single biological replicate were further standardized using a robust version of the Z-score.

bioRxiv preprint doi: https://doi.org/10.1101/2025.06.03.657645; this version posted June 3, 2025. The copyright holder for this preprint (which was not certified by peer review) is the author/funder. This article is a US Government work. It is not subject to copyright under 17 USC 105 and is also made available for use under a CC0 license.

The Z-score values for the same well and plate combination in different biological replicates were then averaged to obtain a Mean Z-score.

Screen hits were identified as genes whose knockout resulted in a mean Z-score either higher than 2.5 or lower than -2.5 for either Number of CENP-C Spots per Cell or Ripley's K clustering score. Nuclei that were either abnormally shaped (solidity < 0.85) or were abnormally small (area < 30 μm^2) indicative of micronuclei were not used for analysis. We also excluded sgRNAs which resulted in high cytotoxicity (Cell Number Z-score < -2.5) or whose Mean Z-score was smaller than the Z-score standard deviation of two replicates.

DAPI and EdU labelling for cell cycle profiling

EdU labelling was performed using a kit (Thermo Fisher Scientific, Cat. No. C10340) as per manufacturer's instructions. Briefly, cells were incubated with 1 ug/mL EdU for 45 minutes before fixation. Fixed cells were permeabilized and blocked as described for IF staining protocol. Next, genome incorporated EdU molecules during replication were fluorescently labelled by performing a click chemistry reaction for 30 minutes at room temperature. Subsequently cells were washed twice with 1% BSA solution in PBST and stained with 5 µg/mL of DAPI solution in PBS at room temperature for 1 hour.

Image analysis for cell cycle profiling

Total nuclear fluorescence of the DAPI and EdU channel was quantified using HiTIPS (Keikhosravi, Almansour et al. 2024). The quantitation data was processed downstream using R packages and log2 transformed DAPI integrated intensity was used to distinguish the G1 and G2/M subpopulations. Similarly, EdU integrated fluorescence intensity was log2 transformed and cells with detectable EdU intensity as S-phase cells. Cells with DAPI intensity either higher than G2 cells (>4N population) or lower than G1 cells (subG1 population) were not analyzed.

Construction of dTAG-SPC24 and NUF2-dTAG cell lines

Candidate guide RNAs targeting the N-terminus of SPC24 and C-terminus of NUF2 were designed using *sgRNA Scorer 2.0* (Chari, Yeo et al. 2017) and CRISPRor (Concordet and

bioRxiv preprint doi: https://doi.org/10.1101/2025.06.03.657645; this version posted June 3, 2025. The copyright holder for this preprint (which was not certified by peer review) is the author/funder. This article is a US Government work. It is not subject to copyright under 17 USC 105 and is also made available for use under a CC0 license.

Haeussler 2018) (**Data S6**). Briefly, candidate guide RNAs were *in vitro* transcribed (IVT) and tested for cutting activity in cells, using an approach previously described (Gooden, Evans et al. 2021). Based on both the highest indel frequency as well as proximity to the point of insertion, candidate 207 was selected for SPC24 and candidates 286 and 289 were selected for NUF2. Oligonucleotides corresponding to these three guide RNAs were phosphorylated, annealed and ligated into either the pX458 backbone (Addgene #48138) (Ran, Hsu et al. 2013) for SPC24 (pJT142) or pDG458 (Addgene #100900) (Adikusuma, Pfitzner et al. 2017) for NUF2 (pMG1040). pSpCas9(BB)-2A-GFP (PX458) was a gift from Feng Zhang (Addgene #48138). Plasmid pDG458 was a gift from Paul Thomas (Addgene #100900).

To generate the homology directed repair (HDR) donors for construction of dTAG-SPC24 and NUF2-dTAG cell lines, first, DNA fragments with 5' and 3' homology arms were synthesized using Twist Biosciences and subsequently cloned into the pGMC00018 using isothermal assembly (Gibson, Young et al. 2009) to generate intermediate constructs pJT147 (SPC24) and pMG1063 (NUF2). Subsequently, the Puromycin-2A-dTAG-3X-FLAG and 3X-FLAG-dTAG-2A-Puromycin cassettes were then PCR amplified from an existing plasmid (contains 3X-FLAG tagged version of dTAG, generated from addgene #91796 or #91793) and then cloned into pJT147 to generate pJT152 and pMG1063 to generate pMG1064, respectively, using isothermal assembly approach. List of oligoes used to generate these constructs are listed (**Data S6**). All plasmids were sequenced completely using nanopore sequencing.

To generate dTAG-SPC24 homozygous knock-in line HCT116 Cas9 cells were co-transfected with two constructs: plasmid pJT142 encoding sgRNAs targeting the SPC24 locus and Cas9, and pJT152 encoding a donor template to introduce dTAG-FLAG and puromycin resistance using Lipofectamine LTX with Plus reagent (Thermo Fisher Scientific, Cat. No. 15338100) as instructed by manufacturer. Similarly, to generate NUF2-dTAG homozygous knock-in line HCT116 cells were co-transfected with pMG1040 encoding sgRNAs targeting NUF2 locus and Cas9, and pMG1064 encoding a donor template to introduce FLAG-dTAG and puromycin resistance. 24 hours after transfection,

bioRxiv preprint doi: https://doi.org/10.1101/2025.06.03.657645; this version posted June 3, 2025. The copyright holder for this preprint (which was not certified by peer review) is the author/funder. This article is a US Government work. It is not subject to copyright under 17 USC 105 and is also made available for use under a CC0 license.

cells were washed with fresh media and grown in fresh media for 24 hours. Next, cells were selected for puromycin resistance by growing them in the presence of 1.5 µg/mL puromycin (Thermo Fisher Scientific, Cat. No. A1113803) for 72 hours. Every 24 hours, old media was replaced with fresh media containing puromycin. Cells were further expanded in presence of puromycin. Depending on the efficiency of transfection cells took 5-7 days to achieve confluence. The cells were then harvested by using trypsin (Gibco, Cat. No. 15050065). A portion of the cells were frozen to generate a polyclonal stock, and the remaining cells were taken forward for single cell cloning.

Single cell cloning

To generate single cell clones, 500 cells were plated on a 15 cm dish and allowed to form colonies in the presence of puromycin. Single colonies appeared in 7-10 days. To isolate single colonies, 20-30 single colonies were selected, and a cloning cylinder was placed around them. The bottom of the cloning cylinder was sealed with grease. For clone isolation, 20 µL trypsin was added to each cylinder and cells from individual colonies were resuspended in separate wells in a 96 well plate to recover single cell clones.

PCR confirmation

Genomic DNA from single cell clones was isolated using the Biorad reagent (Thermo Fisher Scientific, Cat. No. K0512) as recommended. To identify homozygously tagged dTAG-SPC24 clones KG49 (5'AGCTCAGACTTACAGGCGTG3') and KG76 (5'TGATGGTGCTGATGGTTGCA3') primers were used to amplify the genomic region flanking the site of integration at the SPC24 locus. This primer pair is expected to amplify a 2925 bp fragment from the tagged allele and an 1821 bp fragment from the untagged allele. Similarly, homozygously tagged NUF2-dTAG clones were identified by PCR analysis using primer pair KG37 (5’CTGCTTTTCTTCCCCCACTG3’) and KG64 (5’AGAGGCAGCCTTTTCTCTGA3’). NUF2-dTAG alleles produced a 3046 bp amplicon while the untagged allele produced an amplicon of 1943 bp length.

Western blotting

bioRxiv preprint doi: https://doi.org/10.1101/2025.06.03.657645; this version posted June 3, 2025. The copyright holder for this preprint (which was not certified by peer review) is the author/funder. This article is a US Government work. It is not subject to copyright under 17 USC 105 and is also made available for use under a CC0 license.

Two PCR-confirmed single cell clones were used for western blot analysis to validate depletion of dTAG-SPC24 or NUF2-dTAG. Cells were grown in the presence of 1 μM dTAG13 ligand for 3, 6 and 9 hours. Cells grown in the absence of the ligand were grown as control. Protein samples were prepared using Bio-Rad lysis buffer (Bio-Rad, Cat. No. 1610747) as per the manufacturer's instructions. Briefly, 10^6 cells were collected, washed with PBS and lysed in 100 μL lysis buffer (Bio-Rad, Cat. No. 1610747) to isolate proteins. Anti-FLAG monoclonal antibody (Sigma, Cat. No. F3165) was diluted 1:10000 in blocking buffer (5% BSA solution prepared in TBST) was used for detection of dTAG-SPC24 and NUF2-dTAG in western blots. For loading control anti-beta-tubulin antibody (Millipore Sigma, Cat. No. A2228) was diluted 1:25000 in blocking buffer and used in western blots.

siRNA knockdown assay

siRNA knockdown of a select panel of genes identified in the screen was carried out by reverse transfecting siRNAs (**Data S6**, list of siRNAs used). Briefly, 150 nL of 5 μM siRNA stock solution was spotted on the 384 well glass bottom imaging plates using an Echo liquid handler following addition of 20 μL Optimem media (Thermo Fisher Scientific, Cat. No. 31-985-070) using a Multidrop dispenser. Required amount of Lipofectamine RNAiMAX (Thermo Fisher Scientific, Cat. No. 13778075) was added in each well using the Echo liquid handler and incubated for 30 minutes at room temperature to allow siRNA-lipofectamine complex to form. Next, the required number of cells diluted in 20 μLOptimem media containing 20% FBS (Gibco, Cat. No. 10082147) was dispensed into each well. The imaging plate was incubated for 72 hours at 37 °C in a cell culture incubator. Scrambled (Thermo Fisher Scientific, Cat. No. 4390846) and all-star cell death (Qiagen, Cat. No. 1027298) siRNAs were used as controls to optimize the amount of lipofectamine RNAiMax reagent to achieve maximum transfection efficiency with minimum cytotoxicity.

Statistical analyses

Statistical analysis including ANOVA, Tukey HSD test, and t-test with Bonferroni correction was performed using ggpubr (https://github.com/kassambara/ggpubr) and rstatix (https://github.com/kassambara/rstatix/releases) packages in R version 4.3.2.

bioRxiv preprint doi: https://doi.org/10.1101/2025.06.03.657645; this version posted June 3, 2025. The copyright holder for this preprint (which was not certified by peer review) is the author/funder. This article is a US Government work. It is not subject to copyright under 17 USC 105 and is also made available for use under a CC0 license.

Supplementary figures

Fig. S1: Quantification of centromere clustering using CENP-A and CENP-C as centromere markers.

a, Co-staining of HCT116 cells with CENP-A (red), CENP-C (green) and DAPI (gray). Scale bar: 10 µm **b**, Representative image showing segmentation of DAPI stained nuclei (gray) and CENP-C-stained centromere spots (green) in high-throughput imaging data using HiTIPS. Red lines around the DAPI stained nuclei indicate nuclear segmentation, the green circles around CENP-C spots indicate segmentation of centromeres. Zoomed images of the same nucleus are shown with yellow and pink borders indicate before and after spot segmentation was applied. Scale bar: 10 µm. **c**, Quantification of spot count and **d**, clustering score using CENP-A (red) and CENP-C (blue) as centromere markers in HCT116 cells. Values are from two replicates, with at least 2000 cells analyzed for each experimental condition.

Fig. S2. CRISPR knockout screens for centromere distribution phenotypes in HCT116 cells are reproducible.

a, Mean and standard deviation for phenotypic separation between control sgRNAs with individual data points representing mean value per well for number of spots per nucleus and **b**, clustering score in two biological replicates. **c**, scatter plot showing changes in clustering score or **d**, spot count for replicate 1 (x-axis) and replicate 2 (y-axis) in HCT116 cells for each of the 1068 sgRNAs. A linear regression line (gray) was fitted to the data and Pearson's correlation coefficient calculated is indicated at the top left corner of the plot. **e**, scatter plot and linear regression line correlating changes in clustering score (y-axis) and **f,** spot count (y-axis) with nuclear area (x-axis). Pearson's correlation coefficients and corresponding p-values are indicated at the top left corner. Values are from 2 biological replicates. Typically, 200 to 500 cells were imaged for each target gene per experiment.

Fig. S3: Identification of the molecular determinants of spatial centromere distribution in RPE1 cells.

bioRxiv preprint doi: https://doi.org/10.1101/2025.06.03.657645; this version posted June 3, 2025. The copyright holder for this preprint (which was not certified by peer review) is the author/funder. This article is a US Government work. It is not subject to copyright under 17 USC 105 and is also made available for use under a CC0 license.

Changes in spot count (mean Z-score of two replicates, y-axis) and clustering score (mean Z-score of two replicates, x-axis) for each of the 1068 sgRNAs. The most prominent hits were labelled and color coded as in Fig. 2d. Non-hits are colored in gray. Values are from two biological replicates. Typically, 200 to 500 cells were analyzed for each target gene per experiment.

Figure S4: Cell cycle analysis of clustering factor knockdown and their effect on clustering score.

a, Clustering scores of targets (x-axis) after siRNA knockdown in HCT116 cells. Two control siRNAs for siNCAPH2 are in blue and siScrambled is shown in yellow. Upon siRNA knockdown, the clustering score or spot count for the targets (x-axis) labelled in green change in the same direction as in the CRISPR-KO screens and are compared to the mean value for siScrambled as depicted by a horizontal yellow dotted line. Targets in gray disagree with either clustering score or spot count or both parameters compared to data in CRISPR-KO screens. Three separate siRNA were used per target. **b**, Fraction of cells in each cell cycle stage (x-axis) after knockdown of select targets as indicated (y-axis). Individual sub-populations of the cell cycle are color-coded as identified using DAPI and EdU fluorescence intensity measurement, and their percentages are indicated. **c**, Clustering score (y-axis) for select targets (x-axis) at G1 (brown), S (gray) and G2/M (green) stages. Values are from one representative experiment. Typically, 200 to 500 cells were analyzed per gene per experiment.

Fig. S5: Comparative analysis of cell lines confirms common molecular determinants of spatial centromere distribution.

a, 52 genes (black mesh) that are hits in both HCT116 and RPE1 cells. A total of 113 hits were selected in RPE1 cells for either clustering score (pink, 45) or spot count (blue, 87) and 111 hits in HCT116 cells for either clustering score (gold, 89) or spot count (black, 45). White non-shaded areas indicate unique hits in HCT116 (51) and RPE1 (53) cells. Values are from one representative experiment. Typically, 200 to 500 cells were analyzed for each target gene per experiment. **b**, Z-scores (x-axis) of spot count and clustering

bioRxiv preprint doi: https://doi.org/10.1101/2025.06.03.657645; this version posted June 3, 2025. The copyright holder for this preprint (which was not certified by peer review) is the author/funder. This article is a US Government work. It is not subject to copyright under 17 USC 105 and is also made available for use under a CC0 license.

score for the 52 common hits (y-axis) in HCT116 and RPE1 cells. Genes are color coded based on their category as indicated in Fig. 2e.

Fig. S6: Construction and characterization of FLAG-dTAG-SPC24 and NUF2-dTAG-FLAG cell lines.

a, **b**, CRISPR knock-in strategy for homozygous tagging of SPC24 (**a**) and NUF2 (**b**) with the dTAG-FLAG epitope. Horizontal black arrows indicate positions of primers used for PCR confirmation of the tagged allele. **c**, **d**, Representative images of knock-in cell lines expressing FLAG-dTAG-SPC24 (**c**), and NUF2-dTAG-FLAG (**d**) stained with DAPI (gray), CENP-C (green) and FLAG (red). **e**, **f**, Westen blot images showing levels of dTAG-SPC24 (**e**), and NUF2-dTAG (**f**) at indicated times after incubation with dTAG ligands and the relative ratios of dTAG-SPC24 or NUF2-dTAG to tubulin control at the beginning of depletion (0 hr) are indicated below.

Fig. S7: Quantification of cell cycle stages during G1 and mitotic release experiment.

a, Fraction of G1 (blue), S (green) and G2/M (orange) cells were quantified before (0h) and after (6h) release from double thymidine block in the presence or absence of indicated clustering factors. **b**, Fraction of G1 (blue), S (green) and G2/M (orange) cells were quantified before (0h) and after (6h) release from G2/M block in the presence or absence of indicated clustering factors. Values are from one representative experiment containing three technical replicates. Typically, 200 to 500 cells were analyzed per sample.

bioRxiv preprint doi: https://doi.org/10.1101/2025.06.03.657645; this version posted June 3, 2025. The copyright holder for this preprint (which was not certified by peer review) is the author/funder. This article is a US Government work. It is not subject to copyright under 17 USC 105 and is also made available for use under a CC0 license.

Fig. S1

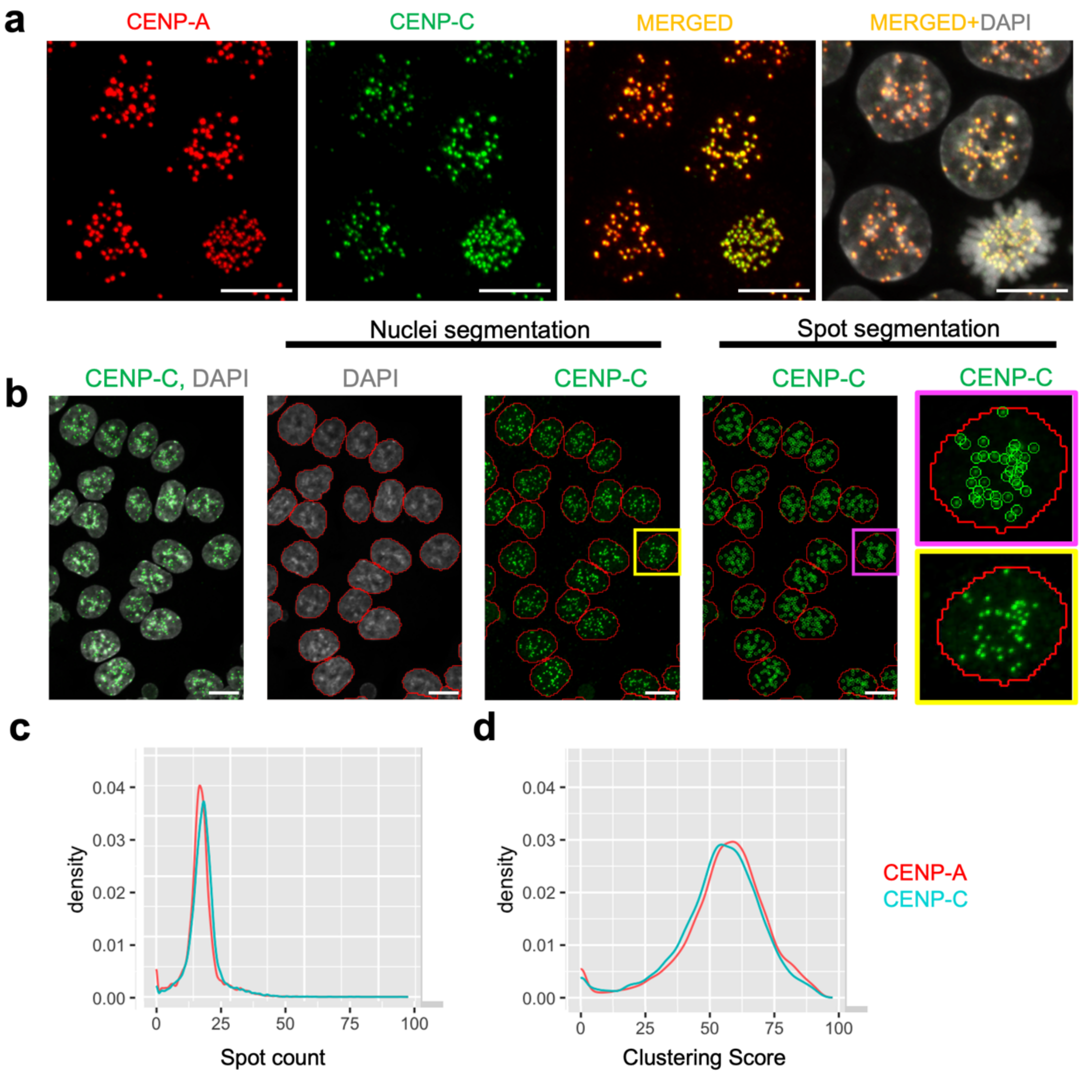

bioRxiv preprint doi: https://doi.org/10.1101/2025.06.03.657645; this version posted June 3, 2025. The copyright holder for this preprint (which was not certified by peer review) is the author/funder. This article is a US Government work. It is not subject to copyright under 17 USC 105 and is also made available for use under a CC0 license.

Fig. S2

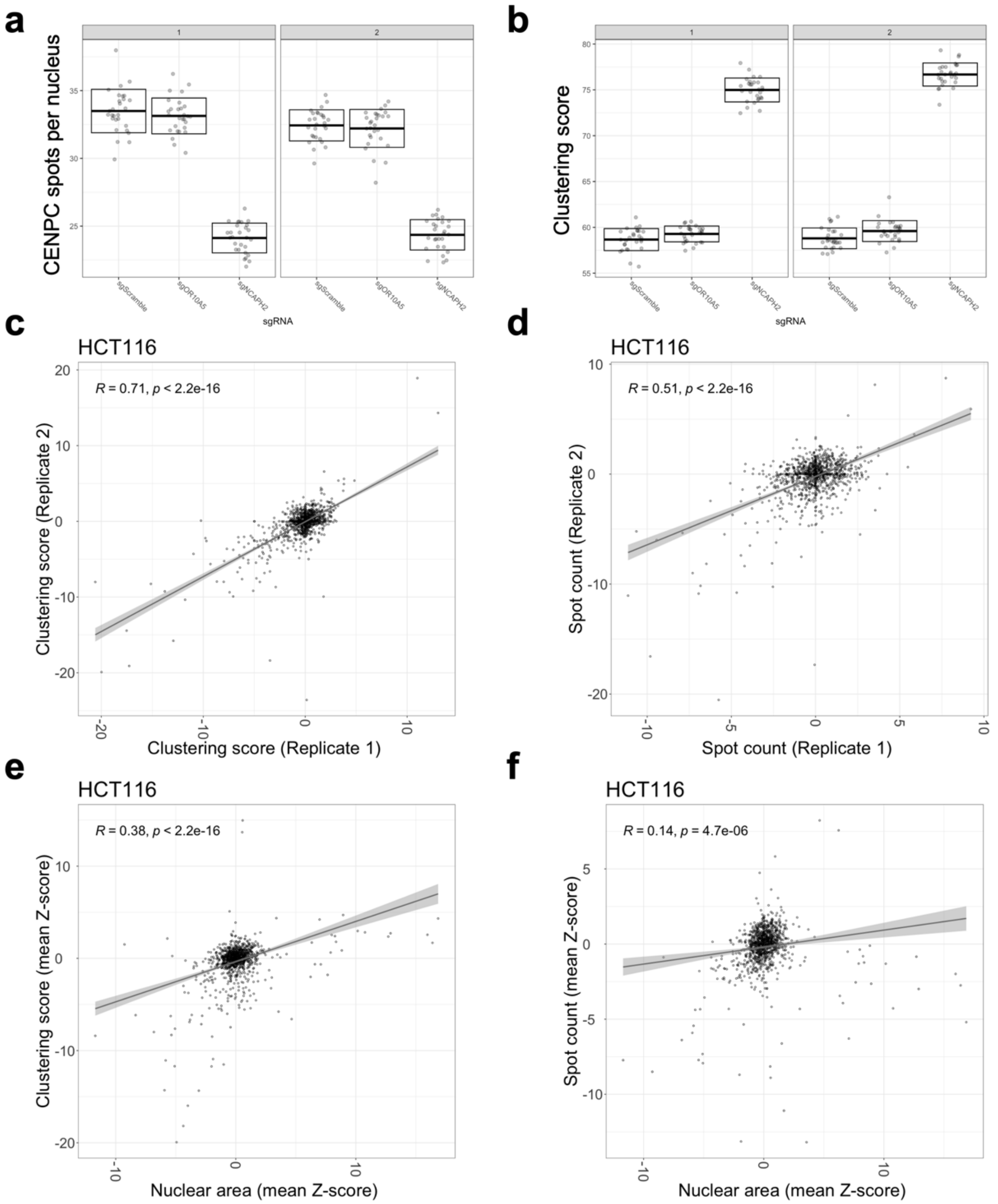

bioRxiv preprint doi: https://doi.org/10.1101/2025.06.03.657645; this version posted June 3, 2025. The copyright holder for this preprint (which was not certified by peer review) is the author/funder. This article is a US Government work. It is not subject to copyright under 17 USC 105 and is also made available for use under a CC0 license.

Fig. S3

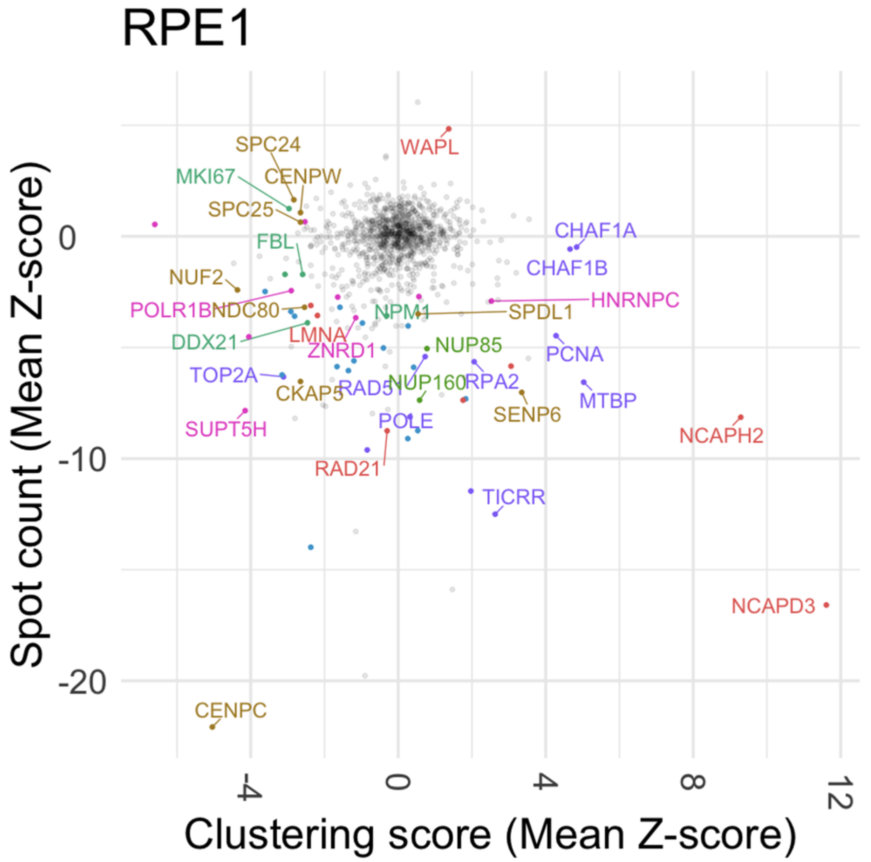

bioRxiv preprint doi: https://doi.org/10.1101/2025.06.03.657645; this version posted June 3, 2025. The copyright holder for this preprint (which was not certified by peer review) is the author/funder. This article is a US Government work. It is not subject to copyright under 17 USC 105 and is also made available for use under a CC0 license.

Fig. S4

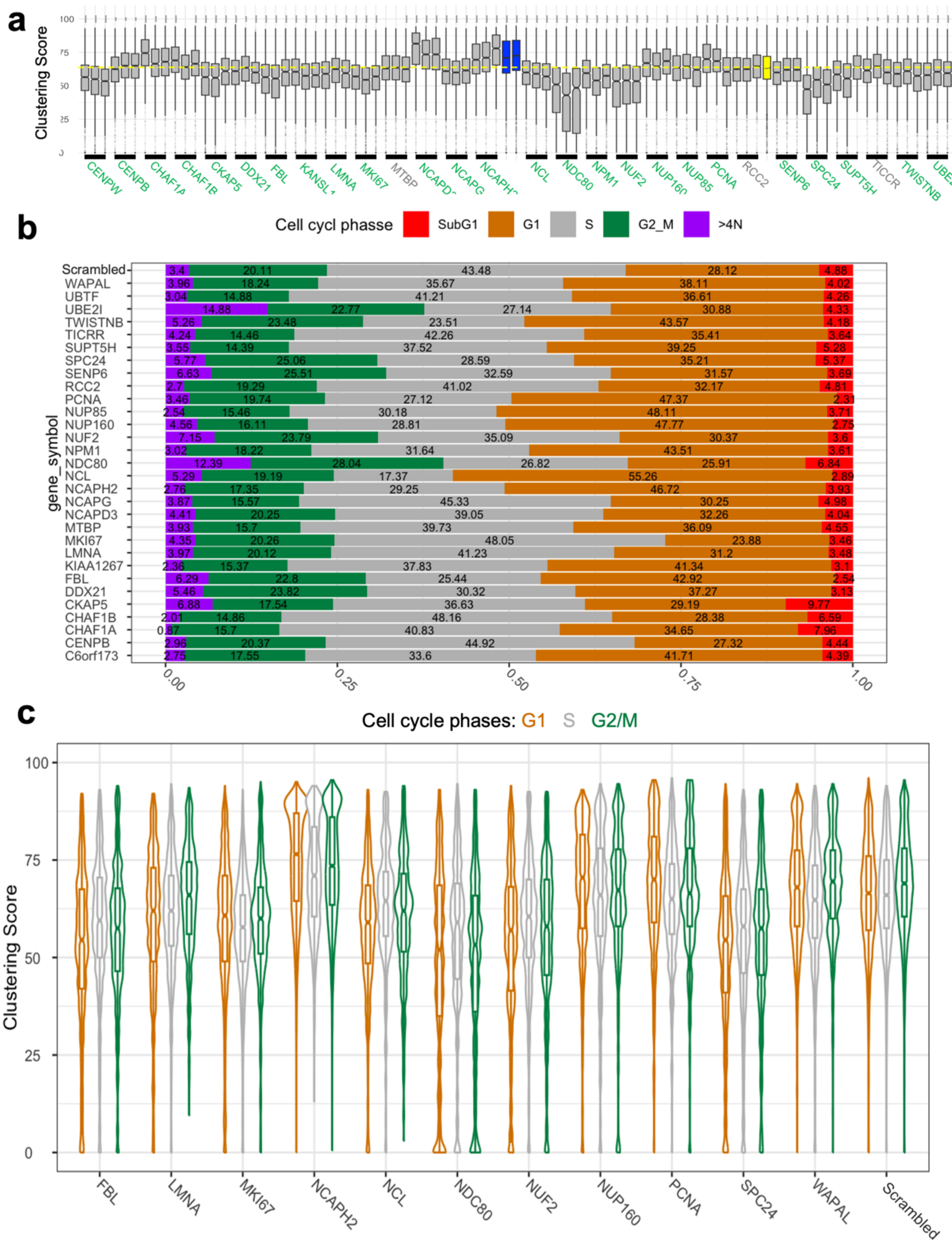

bioRxiv preprint doi: https://doi.org/10.1101/2025.06.03.657645; this version posted June 3, 2025. The copyright holder for this preprint (which was not certified by peer review) is the author/funder. This article is a US Government work. It is not subject to copyright under 17 USC 105 and is also made available for use under a CC0 license.

Fig. S5

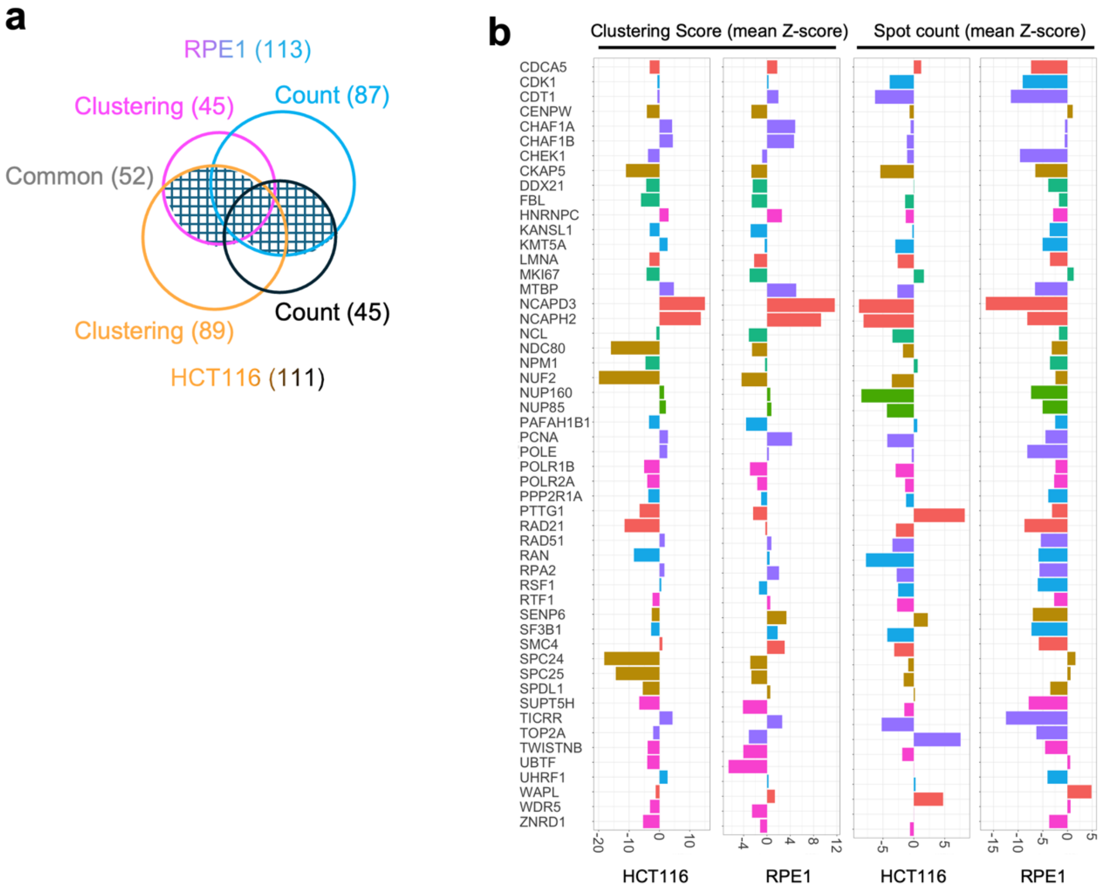

bioRxiv preprint doi: https://doi.org/10.1101/2025.06.03.657645; this version posted June 3, 2025. The copyright holder for this preprint (which was not certified by peer review) is the author/funder. This article is a US Government work. It is not subject to copyright under 17 USC 105 and is also made available for use under a CC0 license.

Fig. S6

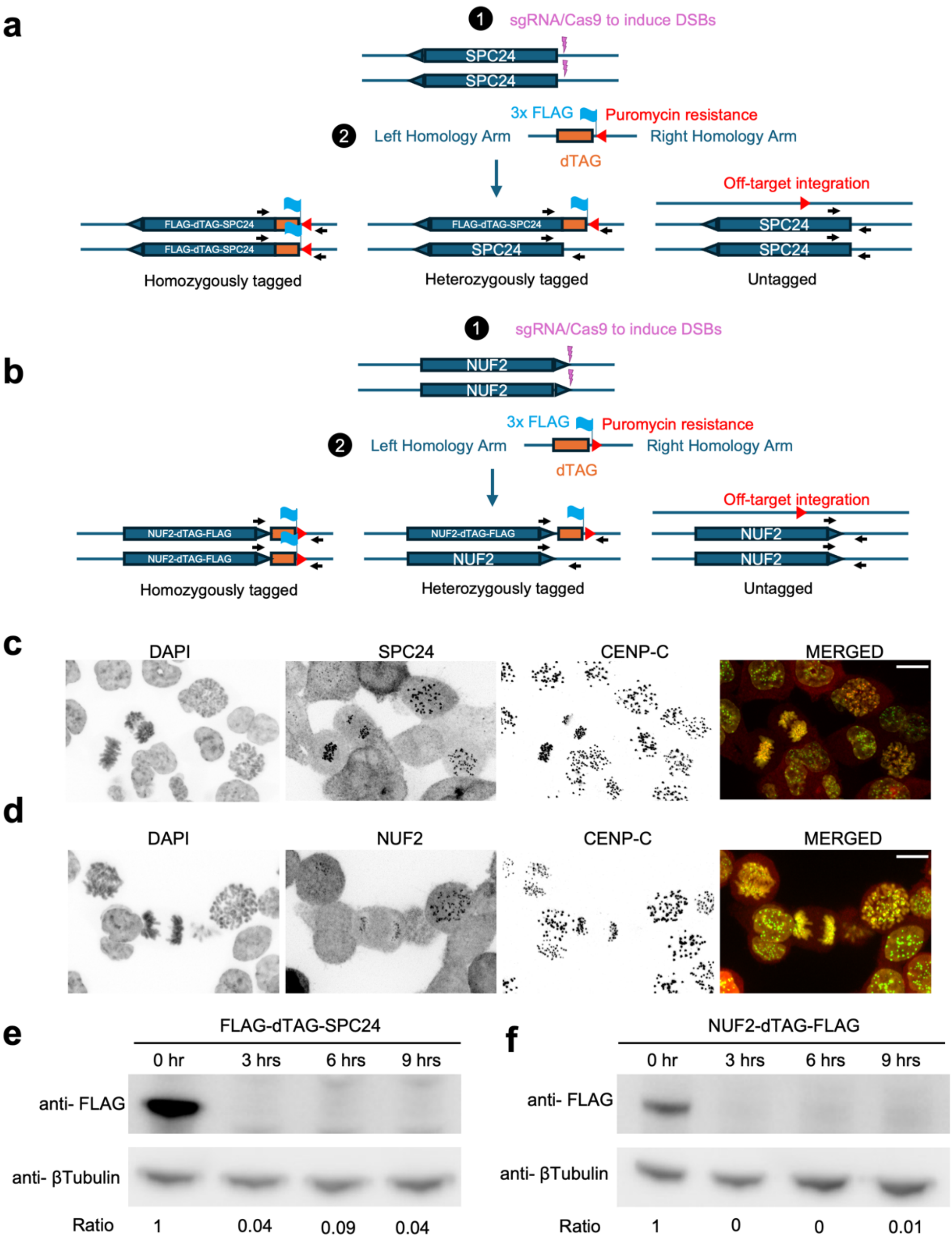

bioRxiv preprint doi: https://doi.org/10.1101/2025.06.03.657645; this version posted June 3, 2025. The copyright holder for this preprint (which was not certified by peer review) is the author/funder. This article is a US Government work. It is not subject to copyright under 17 USC 105 and is also made available for use under a CC0 license.

Fig. S7

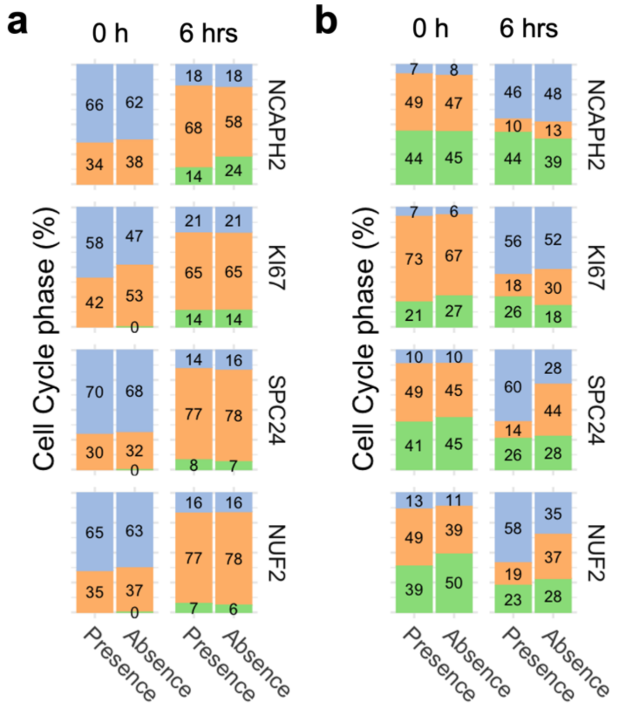

bioRxiv preprint doi: https://doi.org/10.1101/2025.06.03.657645; this version posted June 3, 2025. The copyright holder for this preprint (which was not certified by peer review) is the author/funder. This article is a US Government work. It is not subject to copyright under 17 USC 105 and is also made available for use under a CC0 license.

References

Adikusuma, F., C. Pfitzner and P. Q. Thomas (2017). "Versatile single-step-assembly CRISPR/Cas9 vectors for dual gRNA expression." PLoS One **12**(12): e0187236.

Boutros, M., L. P. Bras and W. Huber (2006). "Analysis of cell-based RNAi screens." Genome Biol **7**(7): R66.

Chari, R., N. C. Yeo, A. Chavez and G. M. Church (2017). "sgRNA Scorer 2.0: A Species-Independent Model To Predict CRISPR/Cas9 Activity." ACS Synth Biol **6**(5): 902-904.

Chen, G. and X. Deng (2018). "Cell Synchronization by Double Thymidine Block." Bio Protoc **8**(17).

Concordet, J. P. and M. Haeussler (2018). "CRISPOR: intuitive guide selection for CRISPR/Cas9 genome editing experiments and screens." Nucleic Acids Res **46**(W1): W242-W245.

Gibson, D. G., L. Young, R. Y. Chuang, J. C. Venter, C. A. Hutchison, 3rd and H. O. Smith (2009). "Enzymatic assembly of DNA molecules up to several hundred kilobases." Nat Methods **6**(5): 343-345.

Gooden, A. A., C. N. Evans, T. P. Sheets, M. E. Clapp and R. Chari (2021). "dbGuide: a database of functionally validated guide RNAs for genome editing in human and mouse cells." Nucleic Acids Res **49**(D1): D871-D876.

Keikhosravi, A., F. Almansour, C. H. Bohrer, N. A. Fursova, K. Guin, V. Sood, T. Misteli, D. R. Larson and G. Pegoraro (2024). "High-throughput image processing software for the study of nuclear architecture and gene expression." Sci Rep **14**(1): 18426.

Keikhosravi, A., K. Guin, G. Pegoraro and T. Misteli (2025). "Simulation and Quantitative Analysis of Spatial Centromere Distribution Patterns." Cells **14**(7).

Pachitariu, M. and C. Stringer (2022). "Cellpose 2.0: how to train your own model." Nat Methods **19**(12): 1634-1641.

Ran, F. A., P. D. Hsu, J. Wright, V. Agarwala, D. A. Scott and F. Zhang (2013). "Genome engineering using the CRISPR-Cas9 system." Nat Protoc **8**(11): 2281-2308.

Vassilev, L. T., C. Tovar, S. Chen, D. Knezevic, X. Zhao, H. Sun, D. C. Heimbrook and L. Chen (2006). "Selective small-molecule inhibitor reveals critical mitotic functions of human CDK1." Proc Natl Acad Sci U S A **103**(28): 10660-10665.
